# Polymeric Nanoparticles with Surface-Anchored Functional Groups as Chelating Agents for Calcium (Ca^2+^) and Magnesium (Mg^2+^) Ions to Inhibit Cellular Interactions

**DOI:** 10.3390/ph18121774

**Published:** 2025-11-21

**Authors:** Lazaro Ruiz-Virgen, Juan Luis Salazar-García, Ismael Arturo Garduño-Wilches, Marlon Rojas-López, Gabriela Martínez-Mejía, Rubén Caro-Briones, Nadia A. Vázquez-Torres, Andrés Castell-Rodríguez, Hugo Martínez-Gutiérrez, José Manuel del Río, Mónica Corea

**Affiliations:** 1 Laboratorio de Investigación en Polímeros y Nanomateriales, ESIQIE, Instituto Politécnico Nacional, Unidad Profesional Adolfo López Mateos, Ciudad de México 07738, Mexico; lazaro1990@hotmail.com (L.R.-V.); juansaga2011@hotmail.com (J.L.S.-G.); gamartinezm@ipn.mx (G.M.-M.); rcaro@ipn.mx (R.C.-B.); 2 Centro de Investigación en Ciencia Aplicada y Tecnología Avanzada, CICATA, Instituto Politécnico Nacional, Calz. Legaría 694, Col. Irrigación, Alcaldía Miguel Hidalgo, Ciudad de Mexico 11500, Mexico; ismael.wilches@gmail.com; 3 Centro de Investigación en Biotecnología Aplicada, Instituto Politécnico Nacional, Ex Hacienda de San Juan Molino, Carretera Estatal Santa Inés, Tecuexcomac–Tepetitla. Km. 1.5, Tepetitla 90700, Mexico; mrojasl@ipn.mx; 4 Escuela Superior de Ingeniería Mecánica y Eléctrica, ESIME, Instituto Politécnico Nacional, Av. Luis Enrique Erro S/N, Unidad Profesional Adolfo López Mateos, Zacatenco, Alcaldía Gustavo A. Madero, Ciudad de Mexico 07738, Mexico; 5 Departamento de Biología Celular y Tisular, Facultad de Medicina, Circuito Interior, Ciudad Universitaria, Universidad Nacional Autónoma de México, Av. Universidad 3000, Ciudad de México 04510, Mexico; nadisva@ciencias.unam.mx (N.A.V.-T.); castell@unam.mx (A.C.-R.); 6 Centro de Nanociencias y Micro-Nanotecnología, IPN-CNMN, Instituto Politécnico Nacional, UPALM S/N Col. Lindavista, Alcaldía Gustavo A. Madero, Ciudad de México 07738, Mexico; humartinez@ipn.mx; 7 Escuela Superior de Física y Matemáticas, ESFM, Instituto Politécnico Nacional, Av. Luis Enrique Erro S/N, Unidad Profesional Adolfo López Mateos, Zacatenco, Alcaldía Gustavo A. Madero, Ciudad de México 07738, Mexico; 8 Posgrado en Ingeniería en Metalurgia y Materiales, ESIQIE, Instituto Politécnico Nacional, Av. Luis Enrique Erro S/N, Unidad Profesional Adolfo López Mateos, Zacatenco, Alcaldía Gustavo A. Madero, Ciudad de México 07738, Mexico

**Keywords:** emulsion polymerization, polymeric nanoparticles, chelating agents, inhibitor agents, drug delivery systems (DDS), breast cancer (BC)

## Abstract

**Background:** Cancer therapeutics development has been a challenge in medical and scientific areas due to their toxicity, limited biocompatibility, and unfortunate side effects. However, despite advances in early detection and the study of novel treatments, the mortality rate for breast cancer remains high, making it a significant global health concern. **Objectives:** In this study, poly(methyl methacrylate) (PMMA) nanoparticles functionalized with acrylic acid (AA), fumaramide (FA), and curcumin (CUR) as chelating and inhibitor agents were synthesized by emulsion polymerization techniques. **Methods and Results:** Comprehensive physiochemical characterization studies based on gravimetry, dynamic light scattering (DLS), electrophoresis, Fourier transform infrared (FT-IR), ultraviolet–visible (UV–Vis) and photoluminescence (PL) spectroscopy, X-ray diffraction (XRD), and scanning electron microscopy (SEM) revealed a pH dependence of nanoparticles that exhibit structural changes upon interaction with calcium (Ca^2+^) and magnesium (Mg^2+^) ions. Calorimetric thermodynamic properties measured by isothermal titration calorimetry (ITC) confirmed chelating coordination and positive cooperativity between the nanoparticles and metal ions. In vitro studies showed the low cytotoxicity of nanoparticles by fibroblast proliferation, and their chelation process was observed by fluorescence microscopy, with the loss of interaction between cells. **Conclusions:** These results suggest that the functionalized nanoparticles have potential in drug delivery systems (DDS) for targeted breast cancer therapies, providing a promising polymer material for more efficient and less toxic treatments.

## 1. Introduction

Cancer arises from the uncontrolled growth and multiplication of abnormal cells within body tissues or organs [1,2]. Currently, this disease is considered the world’s second leading cause of death, according to data reported by the World Health Organization, with an estimated 10 million deaths annually, predominantly affecting women [3]. The most common cancer types affecting the global population are breast, lung, and colon cancer [4,5]. Most breast cancer (BC) cases originate in the connective tissue ducts (ductal carcinoma in situ, DCIS) or lobules (lobular carcinoma in situ, LCIS) and can be classified into four main subtypes: hormonal (luminal A or B), human epidermal growth factor receptor (HER2-positive), and triple-negative breast cancer (TNBC); each one with different standard treatments [4,5,6,7,8]. Tamoxifen, trastuzumab, taxane-based chemotherapy, radiotherapy, and mastectomy are the primary conventional therapies [4,5,6,7,8,9,10,11]. However, these treatments have adverse health effects, including hot flushes, sweating, cardiac issues, neutropenia, diarrhea, fatigue, anemia, thrombosis, and problems with drug distribution specificity [7,12,13,14,15]. Advances in nanoscience, biotechnology, medicine, chemistry, and immunotherapy have led to novel complementary and alternative therapies with promising benefits against breast cancer. For instance, repurposing drugs, metronomic chemotherapy, antibody conjugation systems, targeting BC stem cells, micelles, liposomes, particles, and other nanostructures have been used as drug delivery systems and for breast cancer detection (diagnosis) [10,16,17,18].

Nanoparticles in particular have garnered significant interest in the scientific community due to their physicochemical properties, such as molar mass, chemical reactivity, specific surface area, size, shape, stability, morphology, elasticity, ductility, compatibility, solubility, hydrophilicity, durability, and biodegradability, making them suitable for developing new therapeutic interventions [19,20,21,22,23,24,25]. Polymeric nanoparticles ranging in size from 1 nm to 1000 nm have emerged as release systems to solve difficulties in delivering drugs to tumor cells and reduce the risk of adverse effects on healthy cells. Previous studies have shown that the optimal nanoparticle size is around 100 nm, as they can access regions inside the human body where traditional medical substances are limited [26]. This approach enables the design of the chemical structure, sensitivity, solubility in water, and the amount of drug released using functional groups in particles from synthesis [26,27]. This means attaching some chemical functional segments to the surface area, such as phosphonic (–PO(OH)_2_), thiol (–SH), carboxylic (–COOH), amine (–NH_2_) or amide (–CONH_2_) groups, which are useful for binding several molecules and/or metal ions to form complex structures (chelates) through the coordination of atoms such as nitrogen (N), oxygen (O), and sulfur (S), which possess a pair of free electrons in their structure [27,28,29,30]. In particular, chelating functionalized polymer nanoparticles are organic molecules with two or more electron donor groups that bind coordinately to polyvalent metal ions through the interaction of atoms in their chemical structure [31]. If the functional groups consist of an electron-donating species or can form a resonant structure, the interactions can be stabilized further with (sigma) σ– and/or (pi) π–bond donation [32]. This is attributed to them having differing coordination strengths, such as electronegativity, electron distribution, and number of coordination sites. Acrylic acid is one of the most used monomers for the functionalization of nanoparticles due to its ability to form chelate complexes between the carboxylic group and divalent metal ions; for example, copper (Cu^2+^), cadmium (Cd^2+^), lead (Pb^2+^), zinc (Zn^2+^), nickel (Ni^2+^), cobalt (Co^2+^), calcium (Ca^2+^), and magnesium (Mg^2+^) [33,34]. Fumaramide also leads to the generation of hydrogen bonds due to its conformational chemical structure and affinity toward metal ions and, finally, curcumin has been widely researched because of its antibacterial, metabolic, detoxification, antioxidant, anti-inflammatory, and anticancer properties and the formation of intermolecular bonds with ions of iron (Fe), mercury (Hg), and selenium (Se) [35,36,37]. Specifically, calcium (Ca^2+^) and magnesium (Mg^2+^) ions are the most abundant cations in the human body. They control a diverse and wide range of cellular processes, such as cell cycles, gene transcription, DNA replication, energy metabolism, protein synthesis, bone mineralization, and cytoskeleton activation, and they also function as antioxidant agents and even as an immune response in therapeutic treatments for certain illnesses [38,39,40]. Therefore, calcium and magnesium have equally important roles in regulating cell growth, with magnesium being considered the natural calcium antagonist [40]. Several studies have provided evidence that alterations in Ca:Mg ratios suggest frequently dysregulated cell functions, increasing risk factors for the development of cancer [40]. Both divalent cations are correlated through the TRPM7 protein (transient receptor potential melastatin 7). This gene functions as a flow channel for the transport of calcium and magnesium ions within cells, regulating the permeation of these species during cellular mechanisms [8,40,41]. Adenosine-5′-triphosphate (ATP) is a synthesized biological molecule in the mitochondrial membrane of cells that is required for the cellular energy exchange of all living beings [8,40,42]. In most cases, ATP is coordinated to a divalent cation of Ca^2+^ or Mg^2+^, which interacts with the negative charges of the phosphate groups located in its chemical structure. That means, at high concentrations of calcium or magnesium ions, greater amounts of ATP are required [42]. This fundamental nucleotide carries out essential functions, such as chromosome maintenance, macromolecular transport, cell motility, muscle contraction, nerve impulse propagation, and the synthesis of DNA and RNA. Variations in Ca:Mg ratios also influence mitochondrial performance and normal cellular growth [42]. As a result, the TRPM7 protein and ATP regulate the proportions of calcium (Ca^2+^) and magnesium (Mg^2+^) ions used in the different cellular processes mentioned earlier. If calcium levels rise (high Ca-ATP), serum magnesium contents (lower Mg-ATP levels) are significantly impaired and reduced [8,40,43]. This indicates that calcium inhibits magnesium transport, with one ion regulating the other. High intracellular calcium can indeed affect magnesium influx into cells [7,40]. Notably, calcium is involved in cell replication, and elevated calcium levels can enhance cell multiplication. This can promote the abnormal growth of cancer cells, suggesting a potential relationship between calcium dysregulation, cancer progression, and resistance to the anti-cancer effects of drugs [7,40]. The balance between both species has a significant role in breast cancer prevention and/or in the continued growth of cancer cells [40,44]. Thus, polymeric nanoparticles could be employed to regulate calcium and magnesium ions in the TRPM7 channel and the supply of ATP to combat the growth, invasion, and metastasis of breast cancer cells. Treatments and diagnostic approaches using polymeric nanomaterials are expected to have important benefits for medicine in the future, but the use of functionalized nanomaterials also presents significant challenges, particularly regarding impacts on human health and the environment.

This study reports the synthesis, characterization, and functionalization of four series of polymer-based nanoparticles with surface-anchored functional groups and a core–shell morphology synthesized via emulsion polymerization. The ratios of chelating agents such as acrylic acid (AA), curcumin (CUR), and fumaramide (FA) were changed. The functional properties and the interaction effects of calcium (Ca^2+^) and magnesium (Mg^2+^) ions with the anchored functional groups on the particle surface were analyzed. This research aims to advance the development of original polymeric materials for breast cancer treatment, with potential applications in drug delivery systems (DDS). This is based on the anchoring of functional groups such as acrylic acid, fumaric acid, and curcumin on the surface of the nanoparticles as chelating agents with the purpose of regulating Ca:Mg levels and controlling abnormal cell growth. These innovations could have the ability to enhance the physicochemical behavior, chelation process, release efficiency, efficacy, safety, and biocompatibility of novel delivery technologies in the fields of nanoscience and biomedicine for breast cancer therapy.

## 2. Results and Discussion

### 2.1. Synthesis and the Total Solid Content $\left(T_{s}\right)$ of Polymeric Nanoparticles with Different Ratios of Chelating Agents

**Figure 1:**
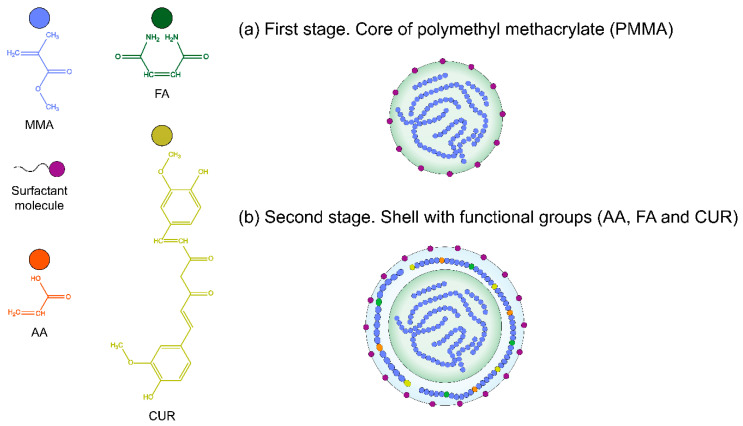
Illustration of the polymerization process of the nanoparticle poly(AA:FA:CUR); (**a**) first stage: Core of polymethyl methacrylate (PMMA); (**b**) second stage: shell with functional groups (AA, FA, and CUR).

The synthesis of particles was conducted in two stages (Figure 1). In the first stage, a core of poly(methyl methacrylate) was prepared, as shown in Figure 1a, while in the second stage, a shell with functional groups was formed, as seen in Figure 1b. Using these methods, four series of methyl methacrylate (MMA)-based polymeric particles functionalized with different proportions of acrylic acid (AA), fumaramide (FA), and curcumin (CUR) as functional agents were created by emulsion polymerization [45,46,47].

The total solid content $\left(T_{S}\right)$ of latex was determined using gravimetric techniques, and the results are shown in Table 1. The obtained latex exhibited average values of 4.28 ± 0.00046 wt.% for all ratios of chelating agents, except for poly(AA:CUR) and poly(FA:CUR), which had average solids values close to 3.48 ± 0.000187 wt.%. This is attributed to the low solubility of curcumin. This contributes to a decrease in the polymerization rate and a low monomer-to-polymer conversion efficiency, thus reducing the solids content in the polymer [48]. On the contrary, those materials with a low content of curcumin or monomers with greater affinity to water (hydrophilicity), such as acrylic acid (AA) and fumaramide (FA), promote high performance in the polymerization process and therefore a higher solids content.

### 2.2. Dynamic Light Scattering (DLS) and Zeta Potential (ζ) of Polymeric Nanoparticles with Different Ratios of Chelating Agents

The dynamic light scattering and electrophoretic mobility methods were used to determine the distributions of particle size and zeta potential (*ζ*) of polymers at 25 °C. The average particle diameters $\left(D_{z}\right)$ and polydispersity index (*PDI*) values were calculated and are summarized in Table 2. The results show that the particle sizes are within the range of 101.2 ± 0.522 <${\;D}_{z}$/nm < 151.8 ± 1.80. In general, these variations in $D_{z}$ are due to the synthesis process and the formulation, concentration, and physicochemical properties of chelating agents of the particles. It is observed that there is a wide range of particle sizes for each series. In particular, poly(AA:FA) particles with 5 wt.% of functional groups have an average size of 121.1 ± 1.82 nm, larger than those with 1 wt.% and 3 wt.% of functional groups. This is because carboxylic (–COOH) and amide (–CONH_2_) groups are highly soluble in water and therefore promote dissolution, polymerization, and anchoring of these functional groups to the shell. Therefore, as the number of hydrophilic groups inside the polymer is higher, bigger particles are obtained. In contrast, as the proportion of hydrophobic segments rises, particle sizes decrease [49,50]. The biggest particle sizes were observed for polymeric systems poly(AA:CUR) and poly(FA:CUR) with 1 wt.% of chelating agents, while polymers with 3 wt.% and 5 wt.% of functional groups had a smaller average size. This behavior is explained by the hydrophobic character and low solubility of curcumin (CUR). However, the presence of carboxylic and amide groups leads to bigger particles being obtained. This corroborates the results obtained by gravimetry, where the materials with high concentrations of curcumin (3 wt.% and 5 wt.%) showed the lowest solids contents (Table 1). Finally, particles of poly(AA:FA:CUR) present slight changes in average particle diameter $\;\left(D_{z}\right)$ caused by the balance generated between the hydrophilic and hydrophobic properties of the chelating agents. In addition, these particles had a polydispersity index (*PDI*) of 1.113 ± 0.004, which indicates a narrow and homogeneous size distribution attributed to the polymerization process and concentration of chelating groups used [51].

Zeta potential (*ζ*) is a physical parameter to evaluate the stability of colloidal dispersions and measures the difference between a particle’s electrokinetic potential and the dispersion medium. This parameter is commonly used to improve and optimize the formulations of emulsions, suspensions, films, coatings, and paints, and predicts particle surface interactions [52]. Nanoparticles with a zeta potential between −10 mV and +10 mV are considered neutral or unstable since the attraction strengths become greater than the repulsion forces, which leads to the disrupt of the dispersion, while those particles with values greater than +30 mV or less than −30 mV are more stable, promoting strong electrostatic repulsions and thus preventing aggregation, flocculation, and/or precipitation [52]. In addition, some factors, such as pH, temperature, concentration, morphology, changes in surface charge during synthesis, and conductivity of the dispersing medium, affect the interparticle interactions [53]. The zeta potential measurements of the obtained polymers revealed values ranging from −27.0 ± 0.224 mV up to −43.8 ± 0.296 mV, indicating a negative surface charge on the particles (Table 3). The average conductivity values during the titration process with CaCl_2_ are provided in the Supplementary Materials in Table S1, as an example. This behavior results in high colloidal stability due to the strong electrostatic repulsions between particles. Negative *ζ* values are attributed to the ionization process of acid groups anchored on the particle surface. In an aqueous medium, carboxyl groups (–COOH) corresponding to acrylic acid (AA) are partially ionized to the carboxylate anion (COO^−^) attributed to the resonance effect by the π–electron of oxygen atom shifting, so hydrogen protons (H^+^) are ionized easily [46,53,54] This phenomenon generates negative repulsive forces among particles, leading to a decrement of zeta potential (*ζ*) and therefore an increment in the materials stability. Otherwise, the amide (–CONH_2_) and phenolic (Ar–OH) groups from fumaramide (FA) and curcumin (CUR), respectively, are in a non-ionized state because the –OH groups (from CUR) and –NH_2_ groups (from FA) are not sufficiently acidic to undergo protonation [46,54]. This suggests that basic functional groups do not have a significant impact on *ζ* response. Additionally, the anionic surfactant used as a stabilizer during polymerization also contributes to the negative surface charge of the particles.

### 2.3. Interaction of Polymeric Nanoparticles with Different Ratios of Chelating Agents and Calcium (Ca^2+^) and Magnesium (Mg^2+^) Ions

#### 2.3.1. Zeta Potential (ζ) of Polymeric Nanoparticles with Different Ratios of Chelating Agents

**Figure 2:**
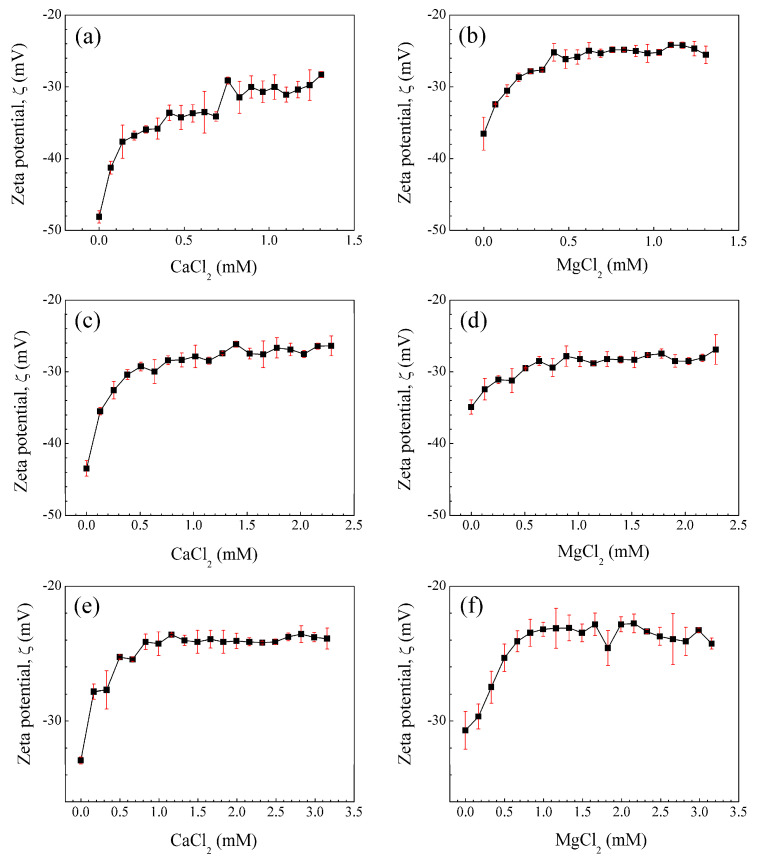
Zeta potential (ζ) of the nanoparticle poly(AA:FA) with (**a**,**b**) 1 wt.%, (**c**,**d**) 3 wt.%, and (**e**,**f**) 5 wt.% of chelating agents as a function of the concentration of calcium chloride (CaCl_2_) and magnesium chloride (MgCl_2_). The vertical red lines (error bars) represent the standard deviation.

The interaction of copolymers with electrolytes was measured by means of zeta potential (*ζ*). The latex samples were titrated with calcium chloride (CaCl_2_) and magnesium chloride (MgCl_2_) solutions. The *ζ* values as a function of electrolyte concentrations for the poly(AA:FA) material with 1 wt.%, 3 wt.%, and 5 wt.% of functional groups are shown in Figure 2, as an example, because all materials had the same behavior (see the Supplementary Materials, Figures S1–S3). In addition, the average conductivity data during the titration process for nanoparticles with CaCl_2_ solutions are provided in the Supplementary Materials in Table S1 as an example.

In general, all curves present a decrement of colloidal stability over the range −48.1 ± 0.832 < *ζ*/mV < −23.1 ± 0.953 for poly(AA:FA), while for other materials, the zeta potential values are within the range of −53.6 ± 3.15 < *ζ*/mV < −22.8 ± 0.702. This is attributed to the interaction of cationic species (Ca^2+^ or Mg^2+^) in the electrolyte solutions with the negatively ionized carboxylate groups (COO^−^) from AA, leading to the adsorption of cations on the surface of particles [55]. This is a result of increasing attractive forces between particles due to the screening effect of cations and the stretching of molecular chains; consequently, *ζ* is increased [53]. This causes the polymeric structural conformation to change abruptly when the divalent cations are attracted by the anions of the particles. Finally, after the amount of salt ions was high enough, the zeta potential (*ζ*) values of the samples were constant due to the saturation of the negatively charged active centers.

In addition, polymer stability also depends on the pH of the dispersion medium and the acid dissociation constant (Ka) related to the degree of ionization of functional groups in the polymeric chains. The values of pH of the polymeric particles before and after electrolyte interaction are shown in Table 4. The average initial and final pH values for the functional polymeric particles are close to 2.59 ± 0.09 and 4.0 ± 0.33, respectively. This pH increment for polymeric systems is attributed to the products formed by the complete dissociation of the electrolyte when interacting with water molecules. For example, acrylic acid (AA) typically has a pKa of 4.1 [56]; however, after the titration process, the pH of the medium remains below this value (pH < pKa), meaning that the carboxylic groups (–COOH) are in a protonated state, avoiding drastic changes in the pH of the solution. Thereby, non-covalent bonds between polymeric chains predominate, and repulsion forces are weak. This behavior is also confirmed by the increment of zeta potential (*ζ*). On the other hand, fumaramide has a pKa close to 14.1, while curcumin shows a broad range of pKa of 8–10 [54,57]. Below these values, they show a stable non-ionized structure and do not contribute to modifying the pH of the environment. Accordingly, the zeta potential (*ζ*) and stability of polymeric nanoparticles are parameters that are clearly dependent on the concentration of ionizable acid groups and the ionic strength of salts [23,53].

#### 2.3.2. Fourier Transform Infrared (FT-IR) and Ultraviolet–Visible (UV–VIS) Spectra of Polymeric Nanoparticles with Different Ratios of Chelating Agents

The presence of functional groups from methyl methacrylate (MMA), acrylic acid (AA), fumaramide (FA), and curcumin (CUR) in the particles was analyzed by Fourier transform infrared spectroscopy (FT-IR).

**Figure 3:**
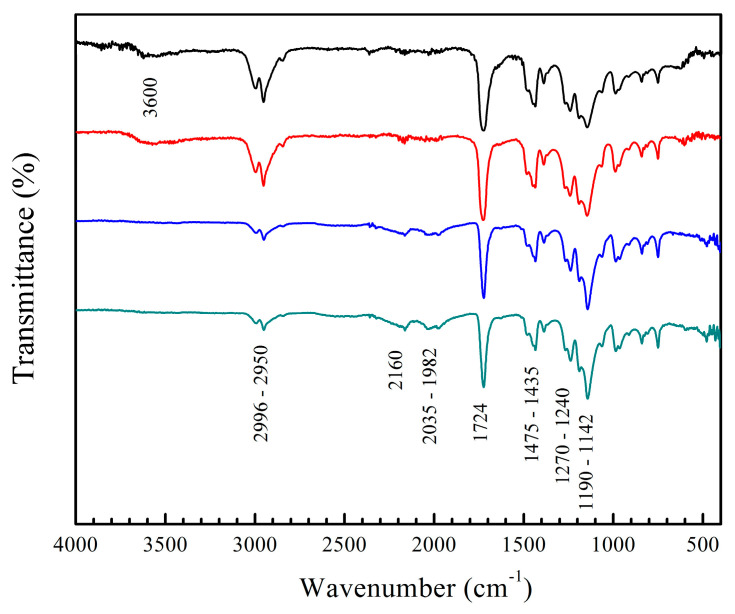
FT-IR spectra of copolymers (black) poly(AA:FA), (red) poly(AA:CUR), (blue) poly(FA:CUR), and (green) poly(AA:FA:CUR) with 5 wt.% of chelating agents.

The spectra of the copolymers with 5 wt.% of functional groups before the titration process are shown in Figure 3, as an example. In general, diverse absorption bands of chemical groups are evidenced and confirm the formation of polymers. The spectra of the AA:FA and AA:CUR copolymers reveal a characteristic peak at 3600 cm^−1^, corresponding to the stretching vibration of hydroxyl groups *v*(OH)_COOH_ of hydrogen bonding in acrylic acid (AA). In addition, bands at 2996 and 2950 cm^−1^ are associated with the stretching oscillation of methyl groups *v*(C–H)_CH3_ from PMMA. Signals at 2160 cm^−1^, 2035 cm^−1^, and 1982 cm^−1^ are assigned to a Fermi resonance effect of the carbonyl group (–C=O) of PMMA [58]. This behavior is a combination of the fundamental stretching vibration of the C=O bond along with the symmetric deformation of the C–H group. An extended band at 1724 cm^−1^ is also attributed to the carbonyl group *v*(C=O) from PMMA [59]. The carbonyl *v*(C=O) binding of pure PMMA typically appears around 1721 cm^−1^, but in these spectra, it is shifted by the copolymerization process with the AA, FA, and CUR monomers [60]. Signals at 1475 cm^−1^ and 1435 cm^−1^ are associated with the bending of the methylene group *δ*(C–H)_CH2_, peaks at 1270 cm^−1^ and 1240 cm^−1^ correspond to the stretching motion of ester group *v*(O=C–O), and absorption bands ranging from 1190 cm^−1^ to 1142 cm^−1^ are attributed to the stretching of the C–O–C bond, *v*(C–O–C) [61]. All these signals are consistent with the methyl methacrylate structure. The amide and phenolic groups of FA and CUR do not appear in the spectra, because the polymerization process was completed, and the signals are possibly overlapped by the broad PMMA bands. The normal absorptions for primary amides *v*(N–H)_NH2_ and stretches of carbonyl groups *δ*(C=O)_CONH2_ in the fumaramide could appear at 3330 cm^−1^ and 1650 cm^−1^, respectively [62,63]. Meanwhile, curcumin (CUR) signals, such as the vibration of hydroxyl group *v*(OH), are detected at 3500 cm^−1^, and a characteristic peak at 3020 cm^−1^ indicates the oscillation of methylene group *v*(C–H), referred to the aromatic ring [64,65,66]. The C–H vibrational frequency appears at 1617–1582 cm^−1^, corresponding to an aromatic overtone, and the peaks ranging from 1484 to 1415 cm^−1^ confirm the presence of the aromatic stretching frequency of the benzene ring [64,65,66]. The carbonyl group δ(C=O), along with the vibration of the aromatic ring *v*(C=C), is observed at 1628 cm^−1^ [64,65,66]. Finally, the peaks appearing at 1346 and 1116 cm^−1^ originate from the vibrational frequencies of C_sp_^2^–O and C_sp_^3^–O bonds, respectively [64,65,66].

**Figure 4:**
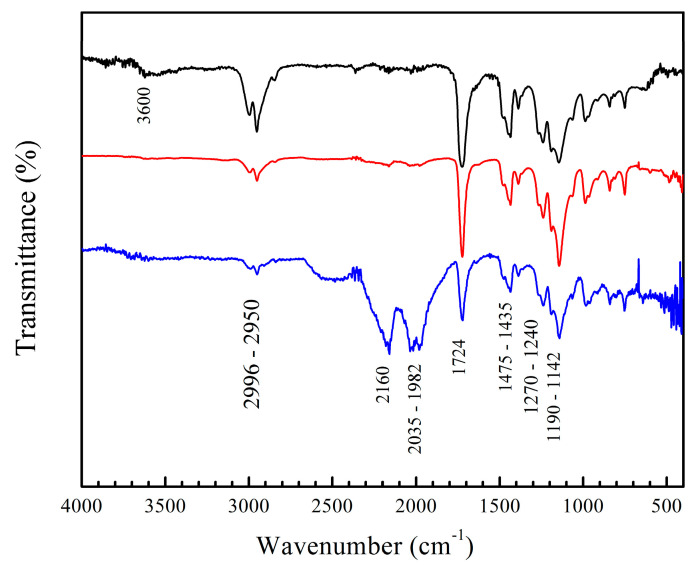
FT-IR spectra of copolymers of poly(AA–co–FA) with 5 wt.% of chelating agents, non-titrated and titrated with calcium chloride (CaCl_2_) and magnesium chloride (MgCl_2_) solutions: (black) poly(AA:FA), (red) poly(AA:FA)–Ca^2+^, and (blue) poly(AA:FA)–Mg^2+^.

The materials were also analyzed by FT-IR after the titration process. The spectra of poly(AA:FA) titrated with CaCl_2_ and MgCl_2_ solutions are shown in Figure 4, as an example. The results indicate a decrease in the intensity of the –OH band (3600 cm^−1^) caused by the disruption of hydrogen bonds coming from the chelation formed among polymers with the metal ions. Additionally, signals from the 2160 cm^−1^ to 1982 cm^−1^ region are increased due to the Fermi resonance phenomena of carbonyl groups (–C=O) that become more intense, especially when magnesium chloride was used. This could be explained by the coordination bond formed between the free electron pairs of carbonyl groups of AA, FA, and MMA with Ca^2+^ and Mg^2+^ ions [67,68,69]. In addition, the Mg^2+^ ion has a smaller solvation radius compared to the Ca^2+^ ion [70]. This allows greater interactions during complex formation due to its larger surface charge density [70]. This is reflected in the increment of the zeta potential (less stability) (Figure 2) along with more pronounced FT-IR bands. (See the Supplementary Materials for Figure S4).

**Figure 5:**
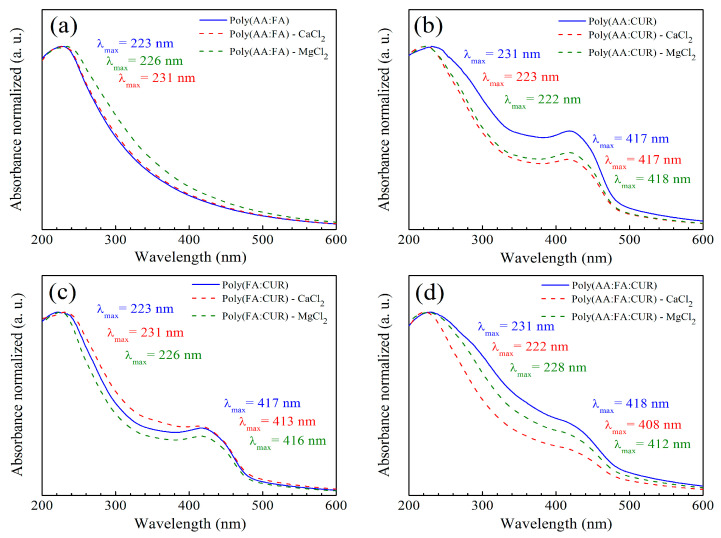
UV–Vis spectra of copolymers (**a**) poly(AA:FA), (**b**) poly(AA:CUR), (**c**) poly(FA:CUR), and (**d**) poly(AA:FA:CUR) with 5 wt.% of chelating agents before and after the titration assessment, where (red dashed line) is -CaCl_2_ and (green dashed line) is -MgCl_2_.

As a complementary technique, functional groups from methyl methacrylate (MMA), acrylic acid (AA), fumaramide (FA), and curcumin (CUR) located on polymeric nanoparticles with 5 wt.% chelating agents were analyzed by ultraviolet–visible (UV–Vis) spectroscopy. The collected data were normalized, and they are presented in Figure 5. A chromophore is a molecule responsible for determining the color of materials that absorb certain wavelengths of visible light, while an auxochrome group modifies the energy bands by increasing the absorption intensity and the maximum wavelength ($\lambda_{max}$) [71,72]. For the polymeric particles before titration, Figure 5a shows the spectrum for poly(AA:FA), with an initial signal at 223 nm, corresponding to the conjugation of chromophore groups of PMMA (O=C–O) and auxochrome species of AA (carboxyl, –COOH) and FA (amide, –CONH_2_). According to the literature, pure PMMA exhibits a typical broadband absorption at 205 nm [73]. This could indicate that the copolymerization process was successfully carried out.

After the titration process, slight wavelength shifts were observed for all systems. This is attributed to changes in the polarity of polymeric solutions due to variations in coordination ligands. For instance, displacements to longer and lower energy wavelengths are referred to as the bathochromic effect (decrease in polarity), while short wavelength shifts and high frequencies are known as a hypsochromic phenomenon (increase in polarity). In agreement with the results, bathochromic wavelength shifts were observed from 223 nm to 226 nm and 231 nm when Ca^2+^ and Mg^2+^ ions were used. This behavior results from the conjugation of divalent cations with the carbonyl (–C=O), carboxylic (–COOH), and amide (–CONH_2_) groups [71]. The spectrum for the copolymer (AA:CUR) is presented in Figure 5b. It is noted that there is an absorbance band at $\lambda_{max}$ = 231 nm, which is attributed to the non-covalent bonds generated between the MMA and AA. In addition, a $\lambda_{max}$ value of 417 nm is assigned to the phenolic group of CUR. Following interaction with electrolytes, the polymeric materials exhibit hypsochromic behavior from 231 nm to 223 nm and 222 nm for polymer-Ca^2+^ and Mg^2+^, respectively. However, bands at 417 nm and 418 nm corresponding to the aromatic group of CUR showed a decrement in absorption due to a charge transfer caused by the complex formation effect, resulting from the electronic transitions in the CUR structure and corresponding to chromophore groups, such as carbonyl (–C=O), and the double bonds in the phenolic group (C=C, Ar-OH), where electrons transition from a bonding π (pi) molecular orbital to an antibonding π* molecular orbital [74,75].

**Figure 6:**
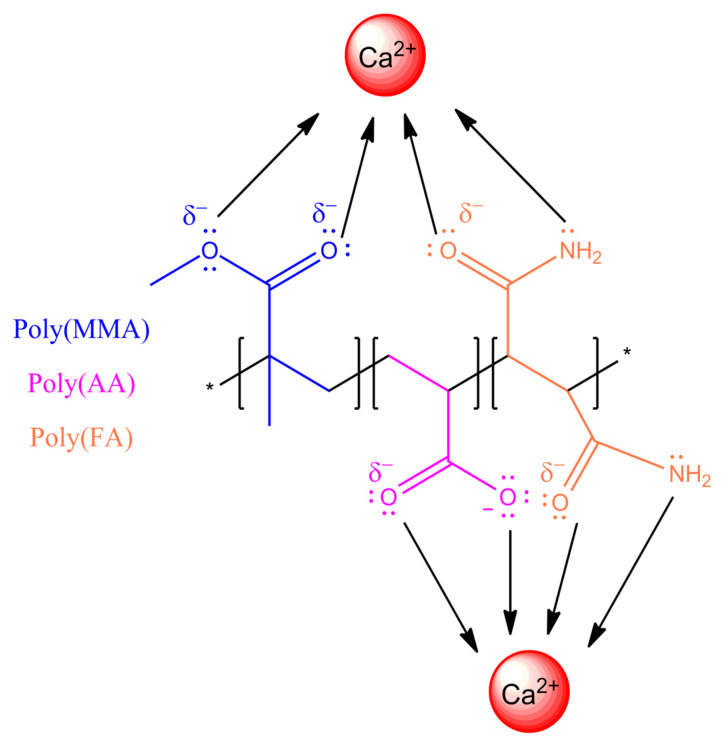
Schematic of interactions and bonding between the copolymer poly(AA:FA) and calcium (Ca^2+^) ions.

The absorption bands of polymer (FA:CUR) are presented in Figure 5c. A peak at 223 nm is observed, corresponding to the combination of the interaction of chromophore species (O=C–O) of MMA with the auxochromes groups (CONH_2_) of fumaramide. After titration with Ca^2+^ and Mg^2+^ ions, bathochromic shifts are present at wavelengths of 231 nm and 226 nm due to interactions with the carbonyl groups. A signal at $\lambda_{max}$ = 417 nm corresponding to the phenolic group of CUR exhibits hypsochromic effects at 413 nm and 416 nm. For the series of nanoparticles of poly(AA:FA:CUR), chromophores of PMMA (–C=O) and auxochromes of AA:FA (COOH:CONH_2_) were identified at 231 nm (Figure 5d). The phenolic (Ar-OH) groups from curcumin are seen at 418 nm. After calcium electrolyte titration, hypsochromic responses from 231 nm to 222 nm and from 418 nm to 408 nm were observed, while for the magnesium solution, displacements of the maximum wavelength ($\lambda_{max}$) decreased from 228 nm and 412 nm. These shifts occur due to chelation of the chemical groups with calcium (Ca^2+^) and magnesium (Mg^2+^) ions, modifying the polymeric structure (Figure 6). The UV–Vis spectra corresponding to the polymers with 3 wt.% and 5 wt.% showed the same behavior.

#### 2.3.3. Scanning Electron Microscopy (SEM) of Polymeric Nanoparticles with Different Ratios of Chelating Agents

**Figure 7:**
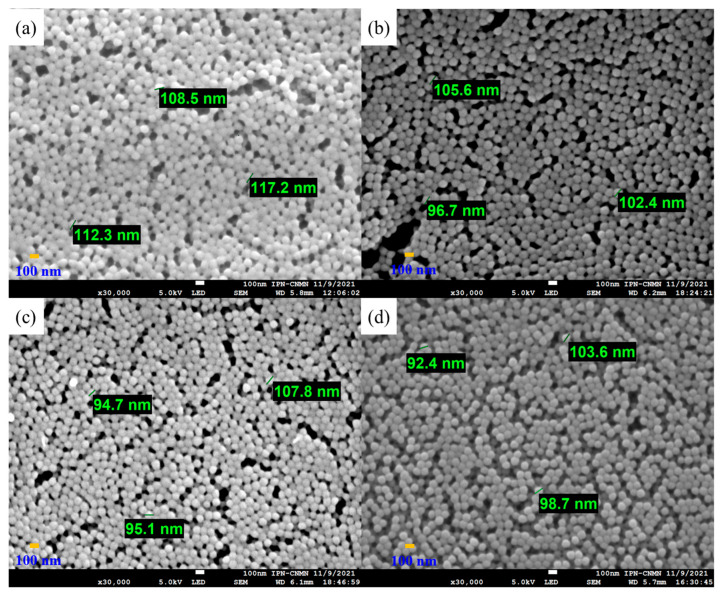
Micrographs of copolymeric nanoparticles with 5 wt.% of chelating agents: (**a**) poly(AA:FA), (**b**) poly(AA:CUR), (**c**) poly(FA:CUR), and (**d**) poly(AA:FA:CUR).

Polymeric particles with 5 wt.% of functional groups were analyzed by SEM to study their morphology, shape, surface, and size distributions (Figure 7). The obtained micrographs show well-defined and spherical particles with an average diameter estimated at around 102.92 ± 7.6909 nm for all combinations of chelating agents. The SEM images present a homogeneous distribution of particles. For all cases, they had no significant changes in morphologies, indicating that the polymers were sufficiently stable. Furthermore, it is clear that the effect of different types of monomers and the distribution of functional groups on nanoparticle shape is negligible. On the other hand, the particle sizes are very similar to the data obtained by the DLS.

**Figure 8:**
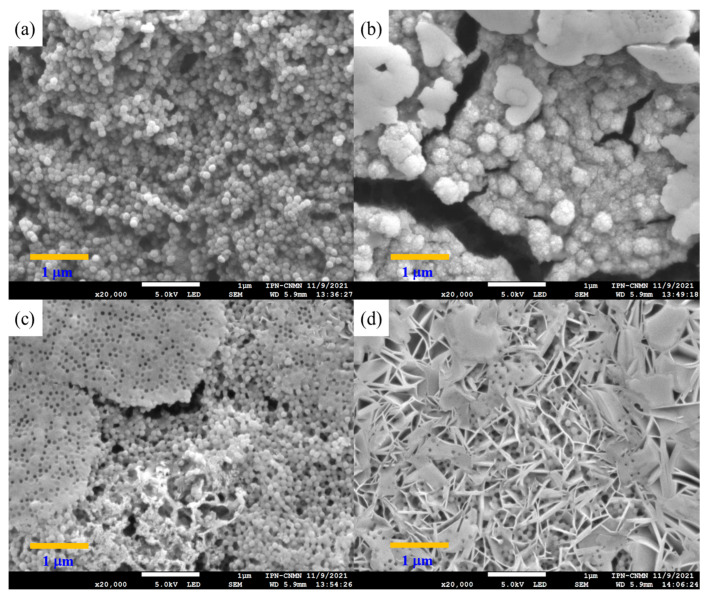
Micrographs of copolymeric nanoparticles with 5 wt.% of chelating agents: (**a**) poly(AA:FA), (**b**) poly(AA:CUR), (**c**) poly(FA:CUR), and (**d**) poly(AA:FA:CUR) titrated with CaCl_2_.

Polymeric nanoparticles with 5 wt.% of chelating agents were titrated separately with calcium chloride (CaCl_2_) and magnesium chloride (MgCl_2_) to observe the conformational changes related to their morphology and chemical structure. Both CaCl_2_ and MgCl_2_ have been used as physical crosslinking agents for the preparation of hydrogels through electrostatic interactions, driven by the attraction between oppositely charged molecules. In an aqueous solution, both salts are dissociated into Ca^2+^ and Mg^2+^ ions [76,77]. The SEM images in Figure 8a–d reveal significantly different size distributions, morphologies, and structures of titrated nanoparticles for all functional group ratios. Figure 8a: poly(AA:FA) and Figure 8b: poly(AA:CUR) polymer particles appear as agglomerates with an irregular surface and non-uniform distribution. This is because the particles lost their configuration as the titration progressed. Likewise, the polymer response is caused by the strong interactions between the hydrophilic groups present (–COOH, AA; –CONH_2_, FA; C=O, MMA; and/or C=O/Ar–OH, CUR) on the particle surface and Ca^2+^ ions, causing an agglomeration process as a consequence of the reduction of repulsive forces between particles and decreasing the negative surface charges of particles [78,79]. These interaction effects are corroborated by the stability evaluation along the titration sequence, where zeta potential (ζ) values were increased to about −20 mV by reducing the negatively charged segments of polymeric chains (Figure 2). Figure 8c for poly(FA:CUR) shows the formation of a three-dimensional porous structure, likely due to the functional groups acting as connection points for physical crosslinking with the Ca^2+^ ion through coordination effects with the carbonyl, amide, and phenolic groups located on the particle surface [80]. This metallic ion induces polymeric network rearrangement by reducing the stiffness and increasing the mobility and flexibility of polymeric chains [81]. Copolymer poly(AA:FA:CUR) exhibited important changes from spherical particles to flat and smooth surfaces (Figure 8d), similar to the behavior exhibited by poly(FA:CUR). Additionally, spherical particles can have a film effect during sample preparation for microscopy. This is because, when a drop of the diluted latex was deposited onto the sample holder and the water was evaporated, the distance between particles decreased, resulting in a closer-packed arrangement of the material and formation of a solid film [82]. This arrangement is more prone to fragmentation, and it is observed in the SEM images for the copolymers (c) poly(FA:CUR) and (d) poly(AA:FA:CUR).

**Figure 9:**
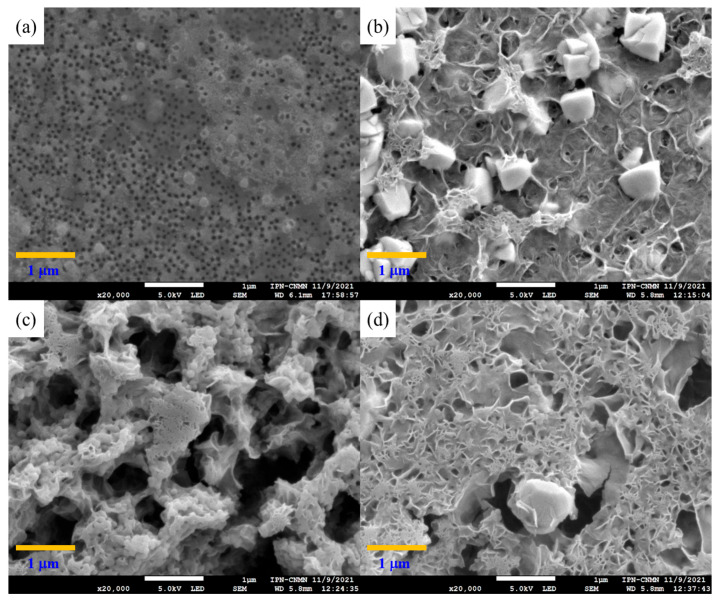
Micrograph of copolymeric nanoparticles with 5 wt.% of chelating agents: (**a**) poly(AA:FA), (**b**) poly(AA:CUR), (**c**) poly(FA:CUR), and (**d**) poly(AA:FA:CUR) titrated with MgCl_2_.

SEM micrographs of the polymeric materials titrated with MgCl_2_ are shown in Figure 9. The observed conformational changes are more noticeable in these systems because the Mg^2+^ ion is smaller than the Ca^2+^ ion and thus has a larger contact surface, resulting in stronger non-covalent associations, as is evidenced in the copolymer poly(AA:FA) (Figure 9a) [70]. This polymeric material displays a fully interconnected porous structure, in which spherical particles are no longer observed by the physical crosslinking through electrostatic interactions between the negative sites of polymer chains and the positive charges of magnesium ions [83]. Images of the particles with AA:CUR and AA:FA:CUR ratios are shown in Figure 9b,d, respectively. The materials appear with a disintegrated and agglomerated structure attributed to the strong interaction with the Mg^2+^ ions, causing restructuring of the chains and leading to a flat and porous structure. Furthermore, the particle size increment could be due to the hygroscopic nature of MgCl_2_; i.e., the capacity to absorb or attract water, which enhances the swelling degree of materials [84]. However, poly(FA:CUR) has aggregations of several spherical particles together with polymeric chains. In this case, the repulsive forces between particles decreased in the presence of a counterion, leading to particle agglomeration through electrostatic attraction. This was confirmed by the zeta potential behavior of the materials [76]. The structural changes in the polymer and their interactions with the Mg^2+^ ions were even more evident than those obtained with the calcium solution, which could be attributed to the Fermi resonance effect observed in the FT-IR results (Figure 4) and through bathochromic and hypsochromic shifts in UV–Vis (Figure 5).

#### 2.3.4. Photoluminescence (PL) Spectra of Polymeric Nanoparticles with Different Ratios of Chelating Agents

**Figure 10:**
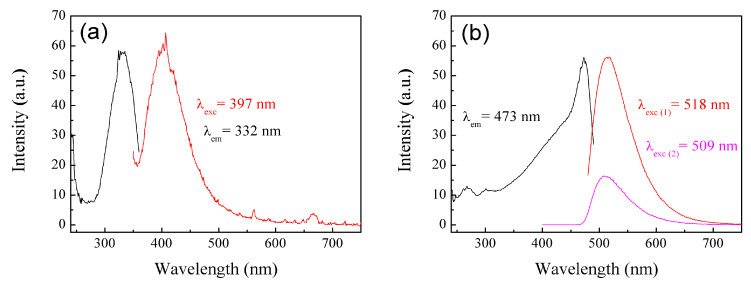
PL spectra of nanoparticles (**a**) poly(AA:FA) and (**b**) poly(FA:CUR) with 5 wt.% of chelating agents.

**Figure 11:**
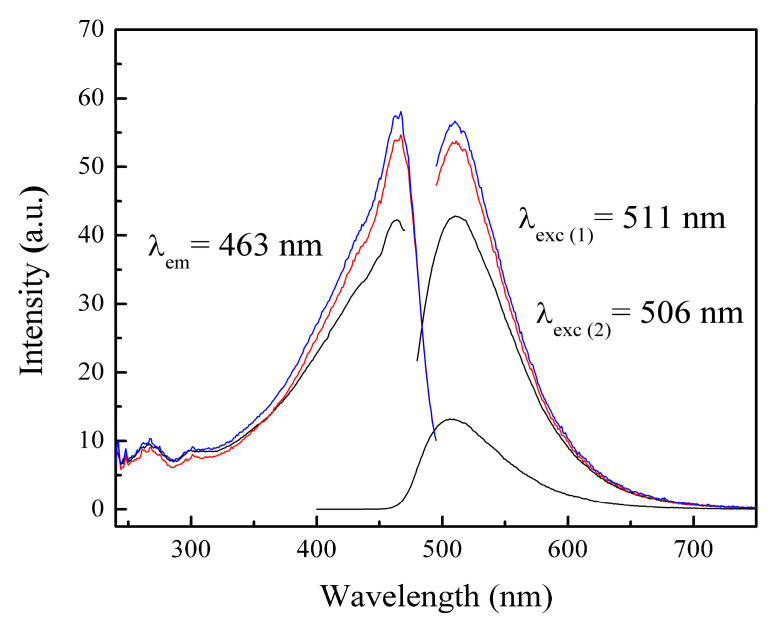
PL spectra of nanoparticles of poly(AA:FA:CUR) with 5 wt.% of chelating agents (black) non-titrated and titrated with (red) CaCl_2_ and (blue) MgCl_2_ solutions.

The materials were analyzed using the photoluminescence technique. Photoluminescence (PL) is a process in which a molecule absorbs a photon in the visible region, exciting one of its electrons to a higher electronic excited state, and then radiates a photon as the electron returns to a lower energy state [85]. If the molecule undergoes internal energy redistribution after the initial photon absorption, the radiated photon is of longer wavelength (lower energy) than the absorbed photon [85]. PL is an emission of ultraviolet, visible, or infrared photons from an electronically excited species [86]. The materials poly(AA:FA) and poly(FA:CUR) with 5 wt.% of chelating agents analyzed by photoluminescence spectroscopy before the titration process are shown in Figure 10a,b, as an example. The obtained experimental emission wavelengths (λ_em_) are 332 nm and 473 nm, respectively. This means that the polymers emit below their corresponding excitation wavelength (λ_exc_). In addition, Figure 11 shows the PL spectra of poly(AA:FA:CUR) with 5 wt.% of chelating agents before and after the electrolyte solution was added. The systems show signals with the same emission and excitation wavelength but different intensities, which is attributed to the chelation process [46]. These results confirm the interactions between the functional groups and the calcium or magnesium ions.

#### 2.3.5. X-Ray Diffraction (XRD) Spectra of Polymeric Nanoparticles with Different Ratios of Chelating Agents

**Figure 12:**
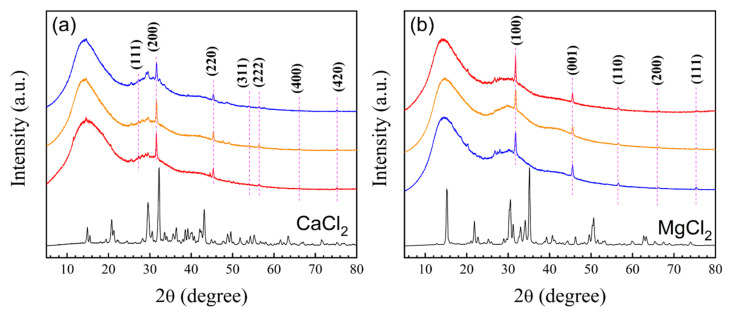
Diffractograms of nanoparticles (red) poly(AA:FA), (orange ) poly(FA:CUR), and (blue) poly(AA:FA:CUR) with 5 wt.% of chelating agents titrated with (**a**) CaCl_2_ (black) and (**b**) MgCl_2_ (black) solutions.

X-ray diffraction (XRD) was used to evaluate the semicrystalline structure of polymeric nanoparticles with 5 wt.% of functional groups before and after titration with electrolytes, and the XRD diffractograms are shown in Figure 12a,b.

For the analysis of the diffractograms, the characteristic diffraction peaks of CaCl_2_, corresponding to a face-centered cubic (FCC) crystal structure, and those of MgCl_2_ associated with a hexagonal close-packed (HCP) arrangement, were used for reference [87,88]. For FCC, the Miller indices (hkl) are either all even or odd (unmixed h, k, l). In contrast, for HCP structures, the indices (hkl) can take any value except when h + 2k = 3n and l is odd [87]. It is important to mention that all peaks (diffraction patterns) that coincided in the three systems (AA:FA, FA:CUR, and AA:FA:CUR) titrated with CaCl_2_ and MgCl_2_ were taken into account for the calculations. To calculate the interplanar spacing (*d*-spacing, in angstroms, Å), Equation (1) was used [88]:

(1) $$d_{hkl}=\frac{\lambda }{2\sin\;\left(\theta \right)}$$

where θ is the angle between the incoming and outgoing beam directions (diffraction), and λ is the wavelength of the incident X-ray beam. The spacing, expressed as 1/d^2^ (Å) for cubic crystal systems, was determined using Equation (2) [88]:

(2) $$\frac{1}{d^{2}}=\frac{h^{2}+k^{2}+l^{2}}{a^{2}}$$

Finally, for polymer–CaCl_2_ systems, the interpretations of basic structures and the calculated indices (hkl) are shown in Table 5.

Meanwhile, the spacing in HCP crystal systems is given by Equation (4) [88]:

(3) $$\frac{1}{d^{2}}=\frac{4}{3}\left(\frac{h^{2}+hk+k^{2}}{a^{2}}\right)+\frac{l^{2}}{c^{2}}$$

As an example, assuming that the first peak is attributed to the (100) plane, in the substitution in Equation (3), a= 3.247 Å, and if the second peak corresponds to the (001) plane, c= 1.990 Å. Thus, *a* and *c* are the crystal lattice parameters. After calculating *a* and *c*, the interplanar spacing (*d_hkl_*) values are in agreement with the peaks obtained from the HCP crystal structure [89]. The *d_hkl_* parameters were calculated separately from the 2θ data and by using Equation (3) to corroborate all of the data. For polymer–MgCl_2_ systems, the interpretations of basic structures and (hkl) indexing are tabulated in Table 6, as an example.

The polymer diffractograms reveal an amorphous region attributed to the non-crystalline nature of PMMA [90]. The corresponding peaks in the complexes formed by the particles with CaCl_2_: 2θ = 27.26°, 31.56°, 45.36°, 54.06°, 56.38°, 66.14°, and 75.16° can be assigned to the (111), (200), (220), (311), (222), (400), and (420) diffraction planes, respectively. These confirm an FCC structure in the systems. Furthermore, when the XRD patterns of the titrated polymers are compared with the CaCl_2_ patterns, a decrement in the intensity of the peaks is observed, where no-covalent bonds are formed and there is interaction by the sharing of electron pairs [83] between calcium ions and the functional groups present in the structure.

Finally, the cubic structure of CaCl_2_ is not intact, as some peaks, such as 2θ = 27.26°, 31.56°, and 45.36°, are slightly shifted, indicating deformation in the physical structure of the CaCl_2_ due to the interactions between the functional groups and Ca^2+^ ions. The diffractograms for the complexes formed between polymeric particles and MgCl_2_ show peaks at 2θ = 31.78°, 45.52°, 56.56°, 66.24°, and 75.34°, corresponding to the diffraction planes (100), (001), (110), (200), and (111), respectively. It is also observed that the intensity of peaks decreased at 15.18°, 21.86°, and 30.54°, combined with a shift in peaks at 31.78°, 45.52°, 56.56°, 66.24°, and 75.34°. These effects are caused by the associations between polymeric particles and Mg ions [91]. The electrolyte-titrated materials exhibit a semicrystalline structure. That is, an amorphous region is attributed to the copolymer, while the crystalline regions are characteristic of the MgCl_2_ [91]. According to the results, the XRD patterns indicate that the magnesium structure also suffers from a slight deformation where shifts in 2θ values suggest distortions in the crystalline lattice of the electrolyte [92], attributed to its incorporation in the polymeric structure.

#### 2.3.6. Isothermal Titration Calorimetry (ITC) of Polymeric Nanoparticles with Different Ratios of Chelating Agents

The intermolecular interactions between the functional groups (AA, FA, and CUR) and the electrolytes were evaluated by isothermal titration calorimetry (ITC) at 25 °C. Because the binding models use concentrations in mol L^−1^, the molecular weights of the polymeric nanoparticles were calculated using the equation described by S. Sajjadi et al. [93], and the obtained values are presented in Table 7. The molecular weights of polymeric nanoparticles were determined in triplicate.

**Figure 13:**
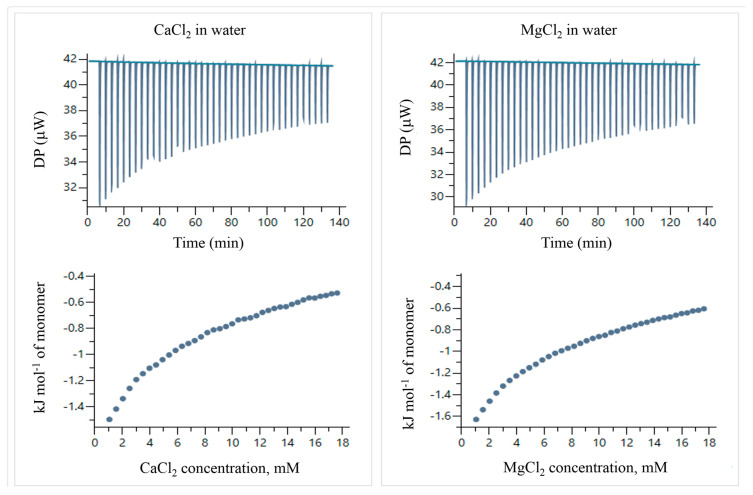
ITC thermograms and temperatures of titration of CaCl_2_ and MgCl_2_ in water at 25 °C.

**Figure 14:**
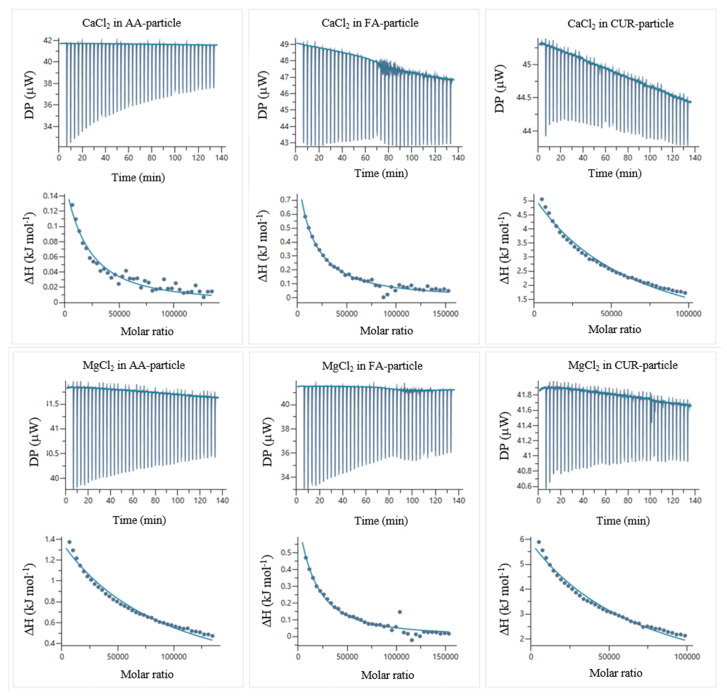
ITC thermograms and corrected temperatures of titration for the AA:CaCl_2_, FA:CaCl_2_, CUR:CaCl_2_, AA:MgCl_2_, FA:MgCl_2_, and CUR:MgCl_2_ systems in water at 25 °C.

Figure 13 shows thermograms of CaCl_2_ and MgCl_2_ solutions titrated in water. Figure 14 presents thermograms of the electrolyte–particle systems in water, and the concentrations used in the experiments are listed in Table 8.

The temperatures of titration were calculated by integrating the peaks of the thermograms using the MicroCal PEAQ-ITC automated software (version 1.22). Figure 14 shows the corrected temperatures, obtained by subtracting the temperatures of titration of the electrolyte solutions in water (Figure 13) from the electrolyte–particle systems.

For these ITC experiments, the polymeric nanoparticles were placed in the titration cell, and the electrolyte solutions were loaded into the syringe. The corrected temperatures of titration as a function of the molar ratio (electrolyte concentration/particle concentration) were fitted to different binding models using the MicroCal PEAQ-ITC automated software (version 1.22). The most accurate fits were obtained using the sequential binding sites model, and the corresponding results are presented in Table 9.

The sequential binding site model [94] considers at least two types of cooperative binding sites in a macromolecule (functionalized polymeric nanoparticles). This means cooperativity requires two or more binding sites that can interact with one another, and these interactions are achieved through conformational changes in the macromolecule [94]. In the studied systems, the binding constant of the first site (K_1_) was lower than that of the second site (K_2_), indicating positive cooperativity [94]. This occurs when the binding of one ligand promotes the subsequent binding of additional ligands beyond the level expected for independent sites [94]. For the interactions between the polymeric nanoparticles and the CaCl_2_ and MgCl_2_ solutions, the metal ions first interact with the particle at a primary binding site, inducing conformational changes on the particle surface that generate a second binding site [94]. These conformational changes could induce drug delivery when the material is applied as a dosing system [95,96], because the negative Gibbs free energy (ΔG°) values confirm the spontaneous nature of the interactions [94,95,96,97,98,99,100,101]. These ΔG values also indicate controlled binding strength, which is beneficial for reversible coordination in drug delivery or chelation systems [94,95,96,97,98,99,100,101]. Furthermore, the thermodynamic parameters suggest that the enthalpic and entropic contributions govern the complexation, depending on the functional group–metal pair. Under acidic conditions, these reversible interactions become advantageous for pH-responsive systems, enabling the controlled release of active compounds or selective metal ion sequestration in acidic environments [94,95,96,97,98,99,100,101].

**Figure 15:**
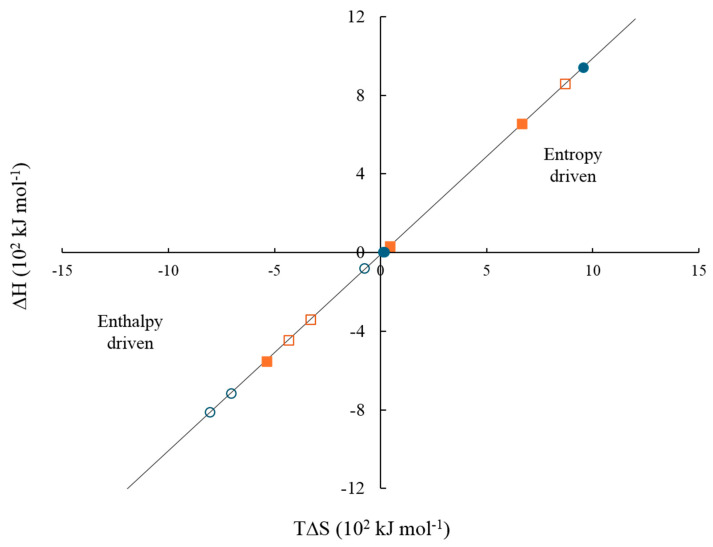
Enthalpy–entropy plot of the interaction between polymeric nanoparticles containing 5 wt.% of chelating agents and electrolytes at 25 °C, considering two cooperative binding sites. (solid orange square) CaCl_2_ site 1, (solid blue circle) MgCl_2_ site 1, (hollow orange square) CaCl_2_ site 2, and (hollow blue circle) MgCl_2_ site 2.

**Figure 16:**
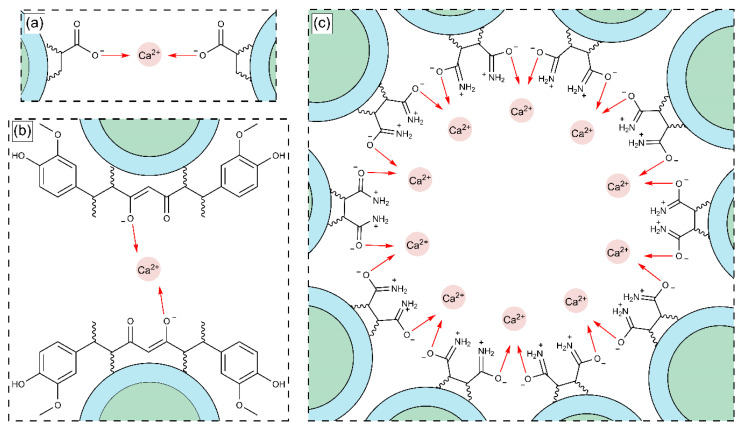
Structural arrangement of the interaction between functional groups: (**a**) AA, (**b**) CUR, and (**c**) FA with calcium ions (Ca^2+^).

Figure 15 shows the enthalpy–entropy plot obtained from the data in Table 9. This qualitative analysis suggests that the first binding site is mainly associated with an entropy-driven process, whereas the second binding site is predominantly enthalpy-driven. This distinction may explain the qualitative differences between the two types of binding sites [94,95,96,97,98,99,100,101]. Based on the results obtained in Table 9, Figure 16a–c illustrates the proposed reaction stoichiometry between the polymers and calcium ions. For AA, one calcium ion (Ca^2+^) is required to form a single interaction site. In the case of FA, an arrangement may exist in which ten amide group molecules interact with ten metal ions. This behavior is consistent with the structures observed by SEM. For CUR, two molecules are necessary per metal ion to achieve one site of interaction. These corroborate the sequential binding sites studied by ITC (Table 9, Figure 14 and Figure 15). Therefore, the thermodynamic findings analyzed above correlate with the proposed interaction mechanisms in Figure 16. Specifically, the stoichiometric ratios derived from these intermolecular interactions highlight differences in spatial arrangement and binding site accessibility, which likely contribute to the observed variation in enthalpy and entropy contributions. Furthermore, the predominance of non-covalent interactions aligns with the compensation trends, suggesting that hydration and the structural organization of the complexes govern the binding energetics [102,103].

#### 2.3.7. In Vitro Cytotoxicity Assay and Cell Viability Analysis of Polymeric Nanoparticles with Different Ratios of Chelating Agents

**Figure 17:**
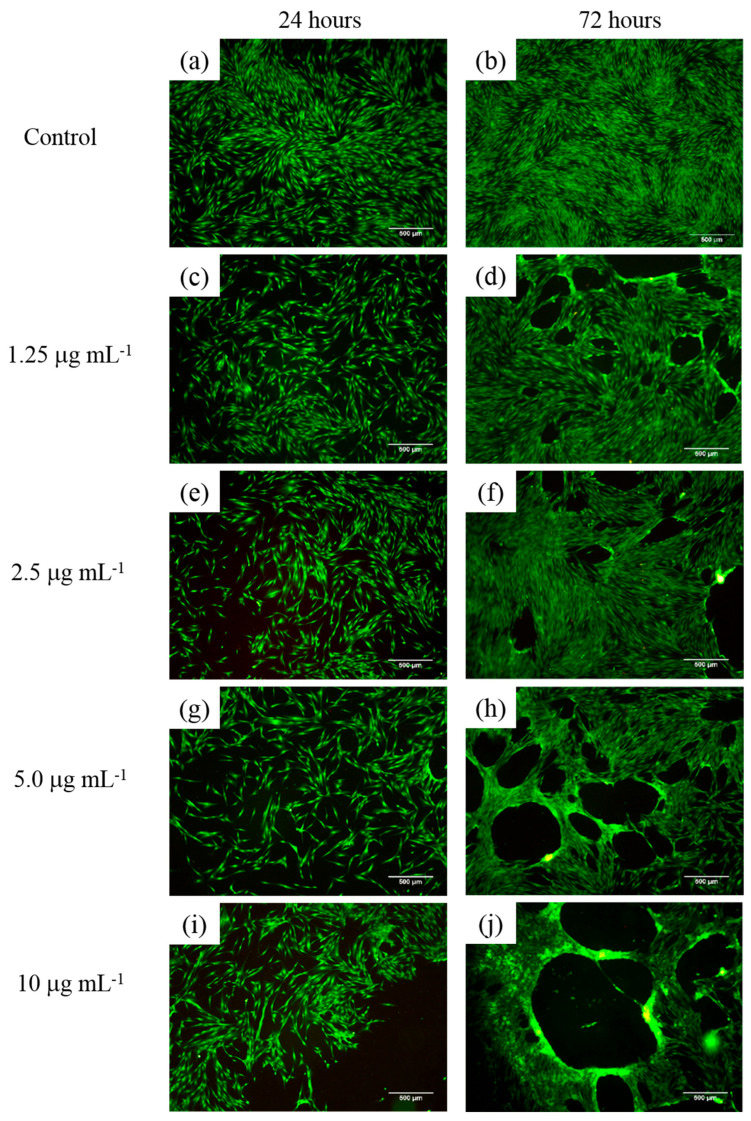
Cell viability assay of human fibroblasts exposed to polymeric nanoparticles (AA:FA:CUR) with 5 wt.% of chelating agents at concentrations of (**a**,**b**) control, (**c**,**d**) 1.25, (**e**,**f**) 2.5, (**g**,**h**) 5, and (**i**,**j**) 10 μg mL^−1^ for 24 and 72 h. Live cells are stained green with calcein, and dead cells are stained red with ethidium homodimer.

**Figure 18:**
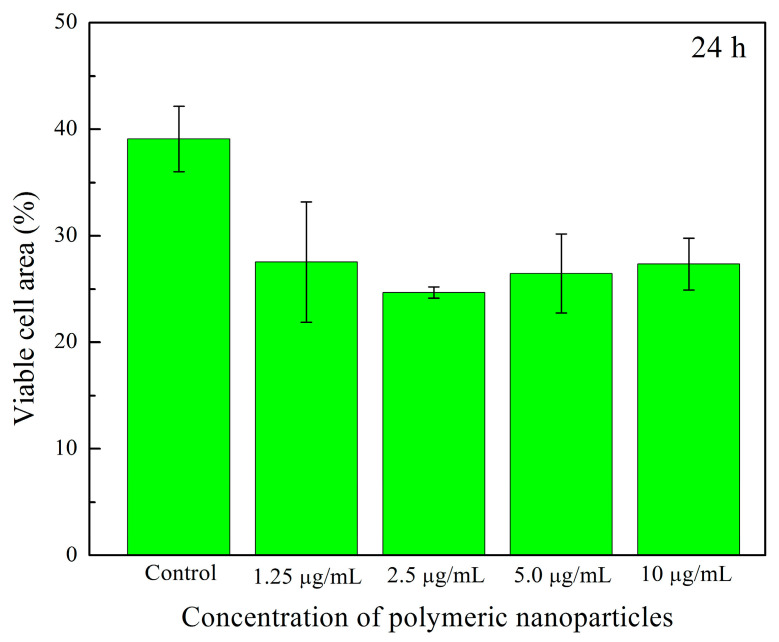
Viable cell area (%) of human fibroblasts exposed to different concentrations (1.25 μg mL^−1^, 2.5 μg mL^−1^, 5 μg mL^−1^, and 10 μg mL^−1^) of polymeric nanoparticles (AA:FA:CUR) with 5 wt.% of chelating agents after 24 h.

**Figure 19:**
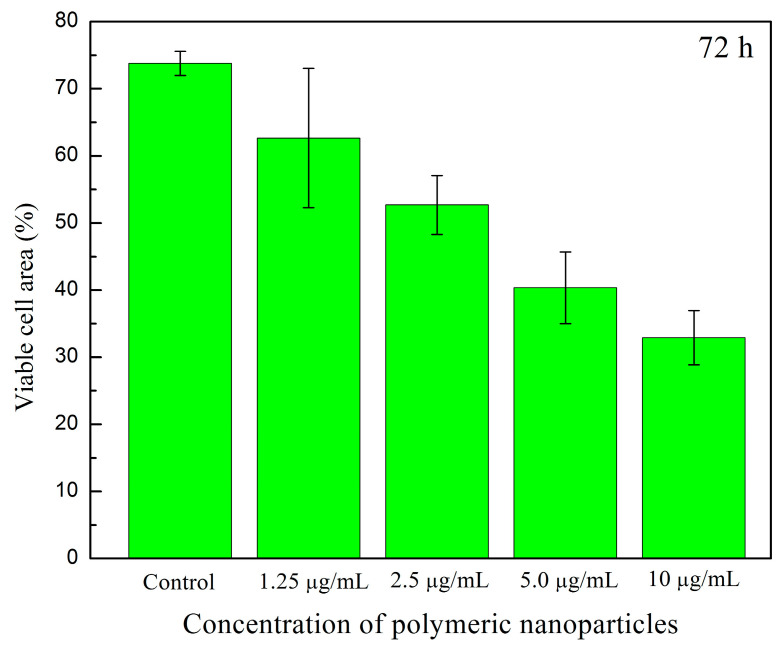
Viable cell area (%) of human fibroblasts exposed to different concentrations (1.25 μg mL^−1^, 2.5 μg mL^−1^, 5 μg mL^−1^, and 10 μg mL^−1^) of polymeric nanoparticles (AA:FA:CUR) with 5 wt.% of chelating agents after 72 h.

Figure 17a–j shows cell viability assays of human fibroblasts interacting with polymeric nanoparticles (AA:FA:CUR) containing 5 wt.% of chelating agents at concentrations of 1.25, 2.5, 5, and 10 μg mL^−1^ after 24 and 72 h, as an example. It is important to observe that nanoparticles containing acrylic acid (AA), fumaric acid (FA), and curcumin (CUR) were used to assess the cytotoxic effects of the three chelating groups on human cells. In general, cell proliferation was observed at all nanoparticle concentrations and interaction times (24 and 74 h). This is indicated by increased cell density compared with the control sample, as shown in Figure 17a,b. Moreover, the cells appear green and exhibit a fusiform morphology, suggesting adhesion to the substrate [104,105,106,107]. This fluorescence behavior indicates that calcein was internalized, metabolized, and reduced by the cells. In contrast, the red fluorescence from ethidium homodimer staining was weak, indicating that the integrity of the cells was maintained and prevented homodimer penetration into the nuclei. These results demonstrate that the polymeric nanoparticles exhibit low cytotoxic effects in human fibroblasts [104,105,106,107]. Specifically, as the concentration of nanoparticles increases, fibroblasts remain viable and maintain complete confluence, as shown in Figure 17c–j. However, after 72 h, an increase in nanoparticle concentration leads to the formation of cell-free areas. This is attributed to the loss of cell–cell interactions caused by the chelation of calcium ions in the medium (from the DMEM solution) by the polymeric nanoparticles [104,105,106,107]. This effect is more evident at a nanoparticle concentration of 10 μg mL^−1^, as shown in Figure 17j. The observed chelation behavior is consistent with the structures shown in SEM images (Figure 8 and Figure 9) and with the structural arrangements suggested by ITC (Figure 16). Figure 18 and Figure 19 show the percentage of the area occupied by viable fibroblasts after 24 and 72 h, respectively. A significant difference was observed between the control and the different nanoparticle concentrations. In Figure 18, no significant differences were found among the four concentrations (1.25, 2.5, 5, and 10 μg mL^−1^). This corroborates the high cell density observed previously (Figure 17). However, at 72 h, the percentage of the area occupied by these junctions prevented cell adhesion and led to a decrease in viable cells compared to the control. Therefore, a significant difference was observed between the control and the 2.5, 5, and 10 μg mL^−1^ concentrations, as shown in Figure 19. This behavior is attributed to the calcium-dependent nature of intercellular junctions and the chelation of calcium ions by the nanoparticles. It can be suggested that the sequestration of calcium disrupts the formation of larger cell-free regions over time and with increasing nanoparticle concentration [104,105,106,107].

These findings are consistent with recent studies on polymeric nanoparticles as inhibitor agents in breast cancer therapy. Recent research has focused on optimizing targeting efficiency, enhancing nanoparticle stability, and overcoming physiological and cellular barriers to achieve superior efficacy [107,108,109].

## 3. Materials and Methods

### 3.1. Materials

The specifications of chemicals used in this study are listed in Table 10. The reagents were used as received, without further purification. The distilled water was double deionized using a Barnstead Micropure water purification system (Thermo Fisher Scientific Inc., Niederelbert, Germany).

### 3.2. Synthesis of Functionalized Polymeric Nanoparticles

**Figure 20:**
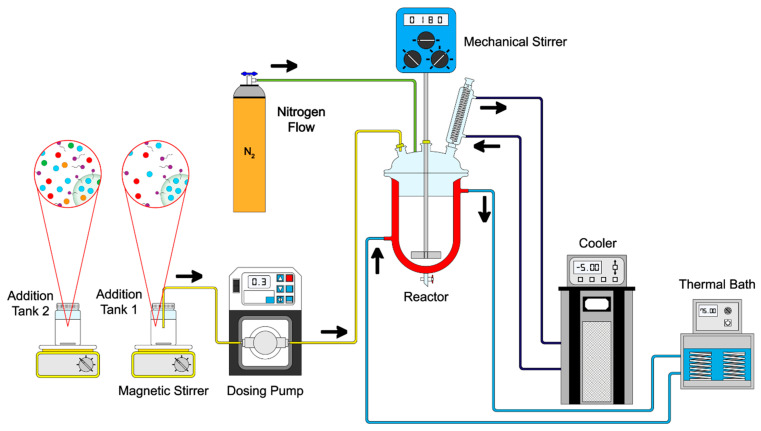
Schematic representation of the emulsion polymerization process of polymeric nanoparticles.

**Figure 21:**
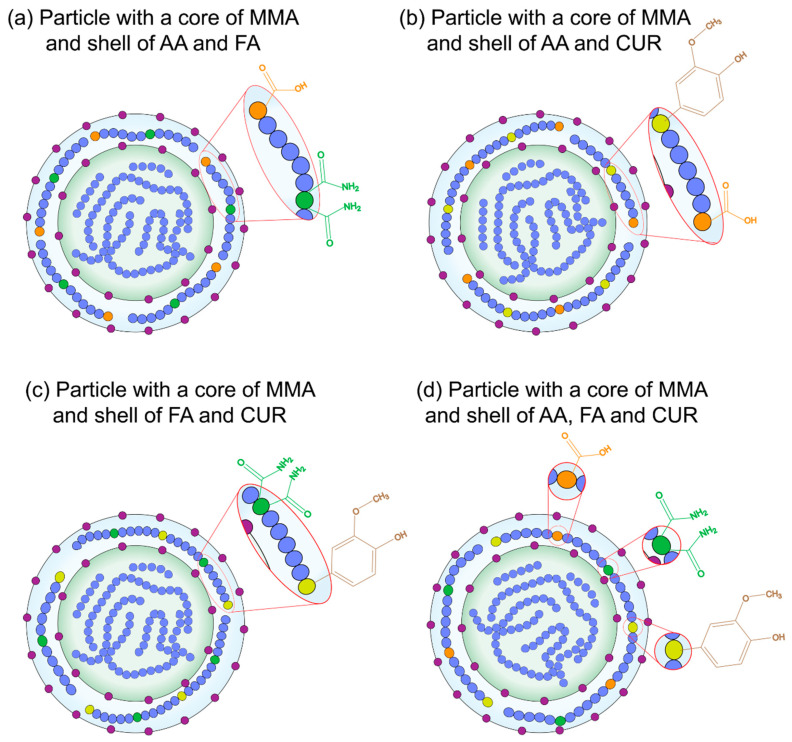
Schematic of the chemical structure of synthesized nanoparticles with a (blue) methyl methacrylate MMA core and a shell of (orange ) acrylic acid, AA, (green) fumaramide, FA, and/or (yellow) curcumin, CUR, where (purple) indicates the surfactant molecules in the polymer chains.

Core–shell polymeric nanoparticles were synthesized via emulsion polymerization techniques in a semicontinuous process in two stages. All polymers were prepared with a total solids content $\left(T_{s}\right)$ of 5 wt.%, and 1 wt.%, 3 wt.%, and 5 wt.% of functional groups on the surfaces of particles. The formulation of the synthesized particles is presented in Table 11 as an example. The polymerization procedure is schematized in Figure 20. In the first stage, a mixture of surfactant (Abex^®^ EP 120, 0.5 wt.%), initiator (sodium persulfate, 2 wt.%), and deionized water was prepared in a flask and then transferred to a 250 mL glass reactor equipped with an external jacket heating system connected to a thermal bath (PolyScience, Niles, IL, USA) with a nitrogen (N_2_) flow of 40 PSI. The emulsion was stirred (180 rpm) using a mechanical stirrer (Heidolph, Schwabach, Germany) until full homogenization at 75 ± 0.1 °C. Once the system temperature was reached, the content of Tank 1 was added to the reactor at 0.3 g min^−1^ using a piston pump (Ismatec^®^ MCP, Wertheim, Germany). In the second stage, a combination of chelating agents, including acrylic acid (AA), fumaramide (FA), and curcumin (CUR), was added as follows: (a) AA:FA, (b) AA:CUR, (c) FA:CUR, and (d) AA:FA:CUR, as shown in Figure 21. Different feed proportions of functional groups or chelating agents (1 wt.%, 3 wt.%, and 5 wt.%) were added to cover the core, according to Table 11 (Tank 2). The second stage was carried out under similar operating conditions to the first stage. Subsequently, a curing stage was performed by increasing the reactor temperature toward the end of the reaction, ensuring complete conversion of the monomers. 

### 3.3. Characterization Techniques

Functionalized polymer nanoparticles with varying ratios of chelating agents were characterized to evaluate their chemical structures, compositions, physicochemical properties, and interaction mechanisms before and after being subjected to a titration process with divalent metal ions.

#### 3.3.1. Gravimetry

The total solid content $\;\left(T_{s}\right)$ of the polymer materials was determined using gravimetric analysis [29]. Five samples were taken from the stock solutions and placed in separate aluminum containers with increments of 0.1 g, ranging from 0.1 g to 0.5 g. The polymeric dispersions were then weighed in an analytical balance (Ohaus Pioneer PA214, Parsippany, NJ, USA) to determine the mass of latex $\;\left(m_{L}\right)$. Subsequently, the polymers were dried in a heating oven (Memmert, Schwabach, Germany) at 60.0 ± 0.1 °C for 12 h to remove water. After drying, the materials were weighed again to obtain the mass of the dry polymer $\;\left(m_{P}\right)$. Finally, the percentage of solids in the polymeric systems was calculated using Equation (4) [45], as follows:

(4) $$T_{s}\;=\;\frac{m_{P}}{\;{\;m}_{L}\;}\;100$$

#### 3.3.2. Dynamic Light Scattering (DLS) and Electrophoresis

The particle size distribution and zeta potential (*ζ*) of the polymer nanoparticles were measured using dynamic light scattering (DLS) and electrophoretic techniques, respectively. Measurements were conducted on a Zetasizer Nano ZSP (Malvern Instruments Ltd., Malvern, UK) equipped with a He-Ne gas laser (λ = 632.8 nm), automatic laser attenuation (transmission 100% to 0.0003%), maximum output power of 10 mW, and measurement angles of ~175°. Before analysis, 0.5 g of each latex was diluted with 15 mL of deionized water to achieve a 1:500 polymer-to-water ratio. A sample was placed in a disposable folded capillary cell (DTS1070). Quadruplicate measurements were performed at 25.0 ± 0.1 °C to obtain the particle size distribution, zeta potential (*ζ*), and pH changes during the interaction between the chelating agents and metal ions. The latex was titrated with calcium chloride (CaCl_2_) and magnesium chloride (MgCl_2_) solutions using an MPT II multi-purpose autotitrator (Malvern Instruments Ltd., Malvern, UK). For this, 0.1 g aliquots of each sample were diluted in 10 mL of deionized water, and the assays were set up over a concentration range of 0.55 mM to 3.15 mM at 25 °C. The collected data were then analyzed to determine stability and calculate the average particle diameters $\;\left(D_{z}\right)\;$ [45,46]. To ensure accuracy and reliability of the data, the measurements were performed in triplicate, following the manufacturer’s protocol [110].

#### 3.3.3. Fourier Transform Infrared Spectroscopy (FT-IR)

FT-IR spectra of polymers were recorded using a Frontier spectrometer (Perkin-Elmer Inc., Waltham, MA, USA) in ATR mode from 4000 to 400 cm^–1^ with a resolution of 4 cm^–1^. All measurements were performed without diluting the sample at 25 °C.

#### 3.3.4. Ultraviolet–Visible Spectroscopy (UV–Vis)

Absorption spectra of all synthesized materials were collected in the 200–1100 wavelength range using an UV–Vis spectrophotometer (Perkin-Elmer Inc., Waltham, MA, USA) at λ = 25 nm. Before starting the analysis, 0.1 mL of the polymeric dispersion was diluted to 100 ppm with deionized water (1:100 ratio) at 25 °C.

#### 3.3.5. Scanning Electron Microscopy (SEM)

The morphology and structure of polymeric particles were observed using a field scanning electron microscope (JSM-7800F, JEOL, Tokyo, Japan) operating under high vacuum at an accelerating voltage of approximately 5 kV, a working distance (W_D_) of 6 mm, and a secondary electron beam. The polymeric material was prepared for analysis by diluting the latex with deionized water at a 1:100 ratio. A drop of the diluted dispersion was placed and dried on the surface of a standard cylindrical copper specimen holder. Then, a thin gold coating was deposited on the sample. Micrographs of non-titrated and titrated polymer particles were obtained using magnifications ranging from 10,000× to 50,000×.

#### 3.3.6. Photoluminescence Spectroscopy (PL)

Photoluminescence (PL) spectra of polymers were recorded at room temperature using a spectrofluorometer FS5 (Edinburgh Instruments, Livingston, UK) in an indirect configuration. The samples were prepared by diluting 0.1 mL of latex in deionized water to obtain a final concentration of 100 ppm (1:100 ratio). The polymeric dispersions were analyzed in a wavelength (λ) range from 240 nm to 750 nm by varying the excitation wavelengths (λ_exc_) to 397 nm, 510 nm, 507 nm, and 511 nm for poly(AA:FA), poly(FA:CUR), poly(AA:FA:CUR), poly(AA:FA:CUR)-Ca^2+^, and poly(AA:FA:CUR)-Mg^2+^ systems, respectively. The emission wavelengths (λ_em_) were also determined.

#### 3.3.7. X-Ray Diffraction (XRD)

X-ray diffraction (XRD) analyses of the titrated polymer materials were performed using a MiniFlex 600 diffractometer (Rigaku, Tokyo, Japan) with a copper X-ray tube (wavelength, λ_Cu_ = 1.5406 Å, 600 W, 40 mA, and 40 kV). The diffraction patterns were recorded over an angular range (2θ) from 2° to 80°. To prepare the samples for XRD analysis, 10 mL of each latex was placed in a crystallizer and dried in a heating oven at 60 °C. The resulting samples were used in powder form.

#### 3.3.8. Isothermal Titration Calorimetry (ITC)

Calorimetry experiments were conducted using a MicroCal PEAQ-ITC automated calorimeter (Malvern Instruments Ltd., Malvern, UK). Polymeric solutions were prepared by diluting latex particles in deionized water to a 2 mM concentration. Calcium chloride (CaCl_2_) and magnesium chloride (MgCl_2_) solutions were prepared in deionized water at 100 mM, maintaining a molar ratio of 1:10 for the functional groups and the electrolyte. For each titration experiment, the polymeric material was put in the cell (200 µL), while the syringe (40 µL) was loaded with the electrolyte solution. The experiments were conducted by establishing 39 injections of 1 µL at 750 rpm, and using a response time of 10 s at 25.00000 ± 0.00012 °C.

#### 3.3.9. Cell Culture

Human fibroblasts were isolated from the surgically removed human skin of healthy individuals who provided informed consent. Cells were seeded at a density of 2.5 × 10^3^ cells per well in 16-well coverglass chamber slides and cultured in high-glucose Dulbecco’s Modified Eagle Medium (DMEM) under standard conditions (37 °C, 5% CO_2_, and 95% humidity). All experiments were performed in triplicate.

#### 3.3.10. In Vitro Cytotoxicity Assay and Cell Viability

The LIVE/DEAD Viability/Cytotoxicity Kit was used to assess cell viability. Human fibroblasts cultured in 16-well chamber slides were treated with nanoparticles (AA:FA:CUR) with 5 wt.% of chelating agents at concentrations of 1.25, 2.5, 5, and 10 μg mL^−1^. Prior to application, the nanoparticles were sterilized using UV radiation for 15 min. Calcein and ethidium homodimer staining were performed after 24 and 72 h of nanoparticle exposure. Untreated wells were used and considered as controls. Fluorescence images were acquired using a fluorescence microscope (Nikon Eclipse, Tokyo, Japan). The percentage of viable cell area was calculated by Image processing using MATLAB (https://www.mathworks.com/products/matlab.html, accessed on 21 October 2025).

## 4. Conclusions

This study presents a novel synthesis approach for polymeric nanoparticles with tailored functional group compositions designed to enhance their physicochemical performance in biomedical applications. The relation between total solids content, particle size, and surface charge demonstrates precise control over colloidal stability and tunable hydrophilic–hydrophobic balance. Notably, the nanoparticles exhibited dynamic chelation with calcium (Ca^2+^) and magnesium (Mg^2+^) ions, revealing a new mechanism for modulating electrostatic interactions and structural organization within polymeric networks. Advanced characterization by UV–Vis, XRD, and ITC confirmed that metal ion coordination induces conformational rearrangements, increased crystallinity, and selective binding through distinct functional sites. Biologically, the nanoparticles maintained non-cytotoxic behavior toward human fibroblasts and promoted cell proliferation at different concentrations, underscoring their biocompatibility. Overall, the synthesized materials represent a significant advancement in the development of metal-chelating polymeric nanoparticles with promising potential as inhibitor agents for breast cancer therapy due to their ability to regulate ionic interactions between cells and promote the controlled transport of calcium and magnesium ions in the TRPM7 protein within the human body.

## Figures and Tables

**Table 1:** Average total solids contents $\;T_{S}\;$ (wt.%) of polymeric nanomaterials with 1 wt.%, 3 wt.%, and 5 wt.% of chelating agents.

Total Solids Content, $T_{s}$ (wt.%)			
Material	1 wt.%	3 wt.%	5 wt.%
Poly(AA:FA)	4.16 ± 0.0002	4.58 ± 0.0006	4.38 ± 0.0002
Poly(AA:CUR)	4.46 ± 0.0006	4.27 ± 0.00008	3.31 ± 0.00003
Poly(FA:CUR)	4.45 ± 0.0006	5.15 ± 0.002	3.64 ± 0.0003
Poly(AA:FA:CUR)	4.48 ± 0.0002	4.25 ± 0.00005	4.26 ± 0.0001

**Table 2:** Average diameters $\;\left(D_{z}\right)$ and polydispersity index (PDI) of polymeric nanoparticles with 1 wt.%, 3 wt.%, and 5 wt.% of chelating agents.

Average Particle Diameter, $D_{z}$(nm), and Polydispersity Index, *PDI*						
Material	1 wt.%		3 wt.%		5 wt.%	
	$D_{z}\;$(nm)	PDI	$D_{z}\;$(nm)	PDI	$D_{z}\;$(nm)	PDI
Poly(AA:FA)	108.9 ± 3.30	1.15	101.2 ± 0.522	1.10	121.1 ± 1.82	1.10
Poly(AA:CUR)	151.8 ± 1.80	1.12	133.0 ± 1.93	1.13	124.8 ± 0.817	1.11
Poly(FA:CUR)	151.4 ± 1.89	1.12	112.8 ± 1.63	1.11	121.1 ± 1.44	1.11
Poly(AA:FA:CUR)	128.2 ± 1.21	1.11	132.2 ± 1.53	1.12	123.3 ± 0.825	1.11

**Table 3:** Zeta potential (ζ) values of polymeric nanoparticles with 1 wt.%, 3 wt.% and 5 wt.% of chelating agents.

Zeta Potential, *ζ* (mV)			
Material	1 wt.%	3 wt.%	5 wt.%
Poly(AA:FA)	−38.7 ± 0.606	−33.7 ± 0.130	−43.8 ± 0.296
Poly(AA:CUR)	−35.5 ± 0.435	−30.1 ± 0.730	−27.0 ± 0.224
Poly(FA:CUR)	−34.0 ± 1.00	−32.4 ± 1.38	−33.3 ± 0.482
Poly(AA:FA:CUR)	−35.1 ± 0.820	−41.5 ± 2.41	−32.9 ± 1.52

**Table 4:** Changes in pH values of polymeric nanoparticles with 1 wt.%, 3 wt.%, and 5 wt.% of chelating agents.

pH						
Material	1 wt.%		3 wt.%		5 wt.%	
	Initial	Final	Initial	Final	Initial	Final
Poly(AA:FA)	2.6 ± 0.014	4.9 ± 1.1	2.5 ± 0.035	4.1 ± 0.19	2.6 ± 0.0071	4.3 ± 0.28
Poly(AA:CUR)	2.6 ± 0.000	3.8 ± 0.21	2.6 ± 0.000	3.8 ± 0.000	2.7 ± 0.0071	3.9 ± 0.11
Poly(FA:CUR)	2.5 ± 0.0071	3.8 ± 0.064	2.5 ± 0.0071	3.8 ± 0.18	2.7 ± 0.0071	4.2 ± 0.099
Poly(AA:FA:CUR)	2.7 ± 0.014	3.8 ± 0.078	2.4 ± 0.049	3.7 ± 0.021	2.7 ± 0.014	3.9 ± 0.078

**Table 5:** Miller indices (hkl) corresponding to the diffraction peaks of polymeric nanoparticles of AA:FA, FA:CUR, and AA:FA:CUR with 5 wt.% of chelating agents titrated with CaCl_2_ solution.

Miller Indices (hkl) Associated with 2θ Values in Cubic Crystal Systems					
2θ (°)	Calculated d_hkl_ (Å)	1/d^2^ (Å^−2^)	1/d^2^/0.09366 (Å^−2^)	Common Factor (CF) Divided by 0.03122	Calculated (hkl)
27.26	3.268	0.09366	1.000	3.000	111
31.56	2.831	0.1247	1.332	3.995	200
45.36	1.997	0.2508	2.677	8.032	220
54.06	1.694	0.3483	3.719	11.16	311
56.38	1.630	0.3764	4.019	12.06	222
66.14	1.411	0.5022	5.362	16.09	400
75.16	1.263	0.6273	6.698	20.09	420

**Table 6:** Miller indices (hkl) corresponding to the diffraction peaks of polymeric nanoparticles of AA:FA, FA:CUR, and AA:FA:CUR with 5 wt.% of chelating agents titrated with MgCl_2_ solution.

Miller Indices (hkl) Associated with 2θ Values in Hexagonal Close-Packed Systems					
2θ (°)	Calculated d_hkl_ (Å)	1/d^2^ (Å^−2^)	1/d^2^/0.1264 (Å^−2^)	(h k l)	Calculated d_hkl_ (Å)
31.78	2.812	0.1264	1.000	100	2.812
45.52	1.990	0.2524	1.997	001	1.990
56.56	1.625	0.3786	2.994	110	1.624
66.24	1.409	0.5035	3.983	200	1.406
75.34	1.260	0.6299	4.982	111	1.258

**Table 7:** Molecular weights of polymeric nanoparticles.

Functional Group in the Particle	Molecular Weight (mol g^−1^)
Acrylic acid (AA)	3.0929 × 10^−8^
Fumaramide (FA)	3.0770 × 10^−8^
Curcumin (CUR)	8.5917 × 10^−8^

**Table 8:** Concentrations used in the experiments of isothermal titration calorimetry (ITC).

Electrolyte–Polymer System	Particles Concentration (μM)	Electrolyte Concentration (M)
CaCl_2_ in AA–particles	0.1584	0.1
CaCl_2_ in FA–particles	0.1393	0.1
CaCl_2_ in CUR–particles	0.05442	0.0250
MgCl_2_ in AA–particles	0.1584	0.1
MgCl_2_ in FA–particles	0.1393	0.1
MgCl_2_ in CUR–particles	0.05442	0.0252

**Table 9:** Thermodynamic parameters and binding constants obtained by fitting the corrected titration heats using the sequential binding sites model.

Systems	K_1_ (M^−1^)	ΔH_1_ (J mol^−1^)	ΤΔS_1_ (J mol^−1^)	ΔG_1_ (J mol^−1^)	K_2_ (M^−1^)	ΔH_2_ (J mol^−1^)	ΤΔS_2_ (J mol^−1^)	ΔG_2_ (J mol^−1^)
AA:CaCl_2_	7.48 × 10^−3^	655	667	−12	1.55 × 10^−2^	−445	−435	−10
FA:CaCl_2_	1.90 × 10^−3^	−554	−538	−16	1.04 × 10^−2^	860	871	−11
CUR:CaCl_2_	9.35 × 10^−3^	31.6	43.2	−11.6	1.89 × 10^−2^	−340	−330	−10
AA:MgCl_2_	3.91 × 10^−3^	4	12	−8	2.06 × 10^−1^	−809	−805	−4
FA:MgCl_2_	5.66 × 10^−3^	4	16.8	−12.8	2.03 × 10^−3^	−80	−76	−4
CUR:MgCl_2_	1.08 × 10^−3^	943	954	−11	1.32 × 10^−2^	−716	−705	−11

**Table 10:** Specifications of chemicals.

Chemical Name	CASRN	Source	Mass Fraction Purity
Methyl methacrylate (MMA)	80-62-6	Sigma–Aldrich, St. Louis, MO, USA	≥0.99 ^a^
Acrylic acid (AA)	79-10-7	Sigma–Aldrich, USA	≥0.99 ^a^
Fumaramide (FA)	627-64-5	ChemCruz, Dallas, TX, USA	≥0.96 ^a^
Curcumin (CUR)	458-37-7	Sigma–Aldrich, USA	≥0.65 ^b^
Calcium chloride (CaCl_2_)	10043-52-4	Sigma–Aldrich, USA	≥0.97 ^a^
Magnesium chloride (MgCl_2_)	676-58-4	Sigma–Aldrich, USA	≥0.98 ^a^
Sodium persulfate (Na_2_S_2_O_8_)	7775-27-1	Sigma–Aldrich, USA	≥0.98 ^a^
Nonylphenol ethoxylate ammonium sulfate (Abex^®^ EP 120)	7732-18-5	Solvay, Houston, TX, USA	— ^b^
Double-distilled water (H_2_O)	—	Mizu Técnica, San Francisco Chimalpa, Mexico	— ^b^

^a^ Reactive grade and ^b^ analytical grade.

**Table 11:** Formulation of polymeric nanoparticles with different acrylic acid (AA) and fumaramide (FA) ratios.

Reagents	Reactor (g)	Tank 1 (g)	Tank 2 (g)		
			1 wt.%	3 wt.%	5 wt.%
Surfactant solution, 0.5 wt.%	0.15	-	-	-	-
Surfactant solution, 3.73 wt.%	-	2.8	1.2	1.2	1.2
Methyl methacrylate (MMA)	-	6.9	3	2.8	2.6
Acrylic acid (AA)	-	-	0.05	0.15	0.25
Fumaramide (FA)	-	-	0.05	0.15	0.25
Initiator solution, 2 wt.%	0.3	1	0.4	0.4	0.4
Distilled water	85	70	30	30	30

## Data Availability

The raw data supporting the conclusions of this article will be made available by the authors on request.

## Supplementary Materials

The following supporting information can be downloaded at: https://www.mdpi.com/article/10.3390/ph18121774/s1, Figure S1: Zeta potential (ζ) of materials Poly(AA:CUR) for (a, b): 1 wt.%, (c, d): 3 wt.% and (e, f): 5 wt.% of chelating agents as function of concentration of calcium chloride (CaCl_2_) and magnesium chloride (MgCl_2_); Figure S2. Zeta potential (ζ) of materials Poly(FA:CUR) for (a, b): 1 wt.%, (c, d): 3 wt.% and (e, f): 5 wt.% of chelating agents as function of concentration of calcium chloride (CaCl_2_) and magnesium chloride (MgCl_2_); Figure S3. Zeta potential (ζ) of materials Poly(AA:FA:CUR) for (a, b): 1 wt.%, (c, d): 3 wt.% and (e, f): 5 wt.% of chelating agents as function of concentration of calcium chloride (CaCl_2_) and magnesium chloride (MgCl2); Figure S4. FT-IR Spectra of copolymers (a) Poly(AA:CUR)-Ca^2+^ and -Mg^2+^, (b) Poly(FA:CUR)-Ca^2+^ and -Mg^2+^, and (c) Poly(AA:FA:CUR)-Ca^2+^ and –Mg^2+^ systems; Table S1. Average conductivity values of polymeric particles with 1 wt.%, 3 wt.% and 5 wt.% of chelating agents during the titration process with CaCl_2_ by DLS technique, as example.
