# Supplementary material for: Polymeric Nanoparticles with Surface-Anchored Functional Groups as Chelating Agents for Calcium (Ca2+) and Magnesium (Mg2+) Ions to Inhibit Cellular Interactions
Source: Pharmaceuticals (Basel). 2025 Nov 21;18(12):1774. doi: 10.3390/ph18121774 (PMC12735866; doi:10.3390/ph18121774)
Supplement: Supplementary file 1 [file pharmaceuticals-18-01774-s001.zip › pharmaceuticals-3881792-supplementary.pdf]

Polymeric Nanoparticles with Surface-Anchored Functional Groups as Chelating Agents for Calcium (Ca^{2+}) and Magnesium (Mg^{2+}) Ions as Inhibit Cellular Interactions.

Lazaro Ruiz-Virgen [a]; Juan Luis Salazar-García [a]; Ismael Arturo Garduño-Wilches [b]; Marlon Rojas-López [c]; Gabriela Martínez-Mejía [a]; Rubén Caro-Briones [a,d]; Nadia Adriana Vázquez-Torres [e]; Andrés Castell-Rodríguez [e]; Hugo-Martínez-Gutiérrez [f]; José Manuel del Río [g,h]* and Mónica Corea [a,*].

[a] Laboratorio de Investigación en Polímeros y Nanomateriales, ESIQIE, Instituto Politécnico Nacional, Av. Luis Enrique Erro S/N, Unidad Profesional Adolfo López Mateos, Zacatenco, Alcaldía Gustavo A. Madero, 07738, Ciudad de México, México; juansaga2011@hotmail.com (J.L.S.G.); lazaro1990@hotmail.com (L.R.V.); gamartinezm@ipn.mx (G.M.M.); rcaro@ipn.mx (R.C.B).

[b] Centro de Investigación en Ciencia Aplicada y Tecnología Avanzada, CICATA, Instituto Politécnico Nacional, Calz. Legaría 694, Col. Irrigación, Alcaldía Miguel Hidalgo, 11500, Ciudad de México, México; ismael.wilches@gmail.com (I.A.G.W.).

[c] Centro de Investigación en Biotecnología Aplicada, Instituto Politécnico Nacional, Ex Hacienda De San Juan Molino, Carretera Estatal Santa Inés, Tecuexcomac–Tepetitla. Km. 1.5, Tepetitla, 90700, Tlaxcala, México; mrojasl@ipn.mx (M.R.L.).

[d] Escuela Superior de Ingeniería Mecánica y Eléctrica, ESIME, Instituto Politécnico Nacional, Av. Luis Enrique Erro S/N, Unidad Profesional Adolfo López Mateos, Zacatenco, Alcaldía Gustavo A. Madero, 07738, Ciudad de México, México; rcaro@ipn.mx (R.C.B).

[e] Departamento de Biología Celular y Tisular, Universidad Nacional Autónoma de México, Facultad de Medicina, Circuito Interior, Ciudad Universitaria, Av. Universidad 3000, 04510, Ciudad de México, México; nadisva@ciencias.unam.mx (N.A.V.T.); castell@unam.mx (A.C.R.).

[f] Centro de Nanociencias y Micro-Nanotecnología, IPN-CNMN, Instituto Politécnico Nacional, UPALM S/N Col. Lindavista, Alcaldía Gustavo A. Madero, 07738, Ciudad de México, México; humartinez@ipn.mx (H.M.G.).

[g] Escuela Superior de Física y Matemáticas, ESFM, Instituto Politécnico Nacional. Av. Luis Enrique Erro S/N, Unidad Profesional Adolfo López Mateos, Zacatenco, Alcaldía Gustavo A. Madero, 07738, Ciudad de México, México.

[h] Posgrado en Ingeniería en Metalurgia y Materiales, ESIQIE, Instituto Politécnico Nacional, Av. Luis Enrique Erro S/N, Unidad Profesional Adolfo López Mateos, Zacatenco, Alcaldía Gustavo A. Madero, 07738, Ciudad de México, México.

*Correspondence: mcoreat@yahoo.com.mx, mcorea@ipn.mx (M.C.); jdelriog@ipn.mx, jm.delrio.garcia@gmail.com (J.M.d.R.).

Supplementary material

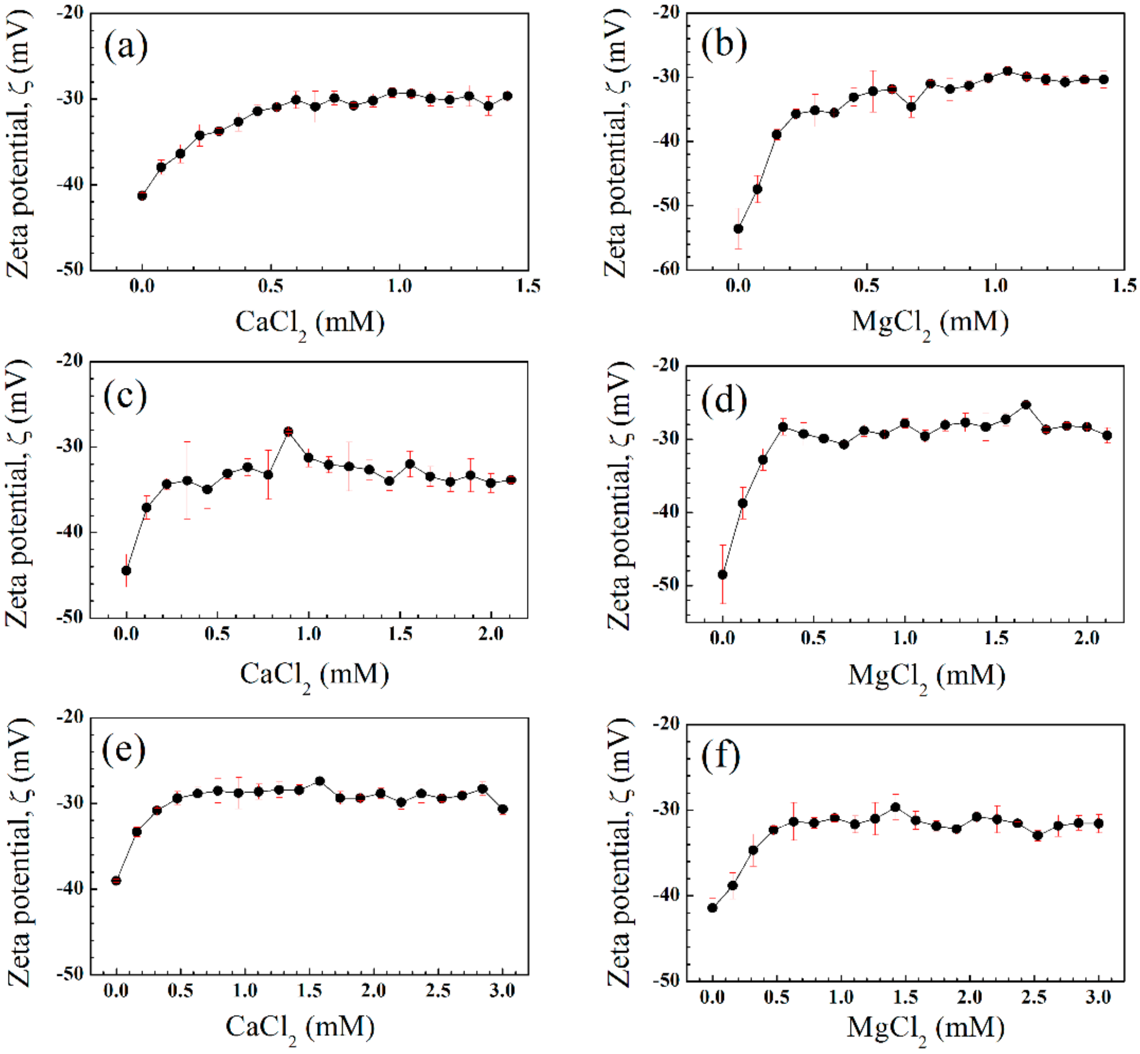

Figure S1. Zeta potential (ζ) of materials Poly(AA:CUR) for (a, b): 1 wt.%, (c, d): 3 wt.% and (e, f): 5 wt.% of chelating agents as function of concentration of calcium chloride ($CaCl_2$) and magnesium chloride ($MgCl_2$).

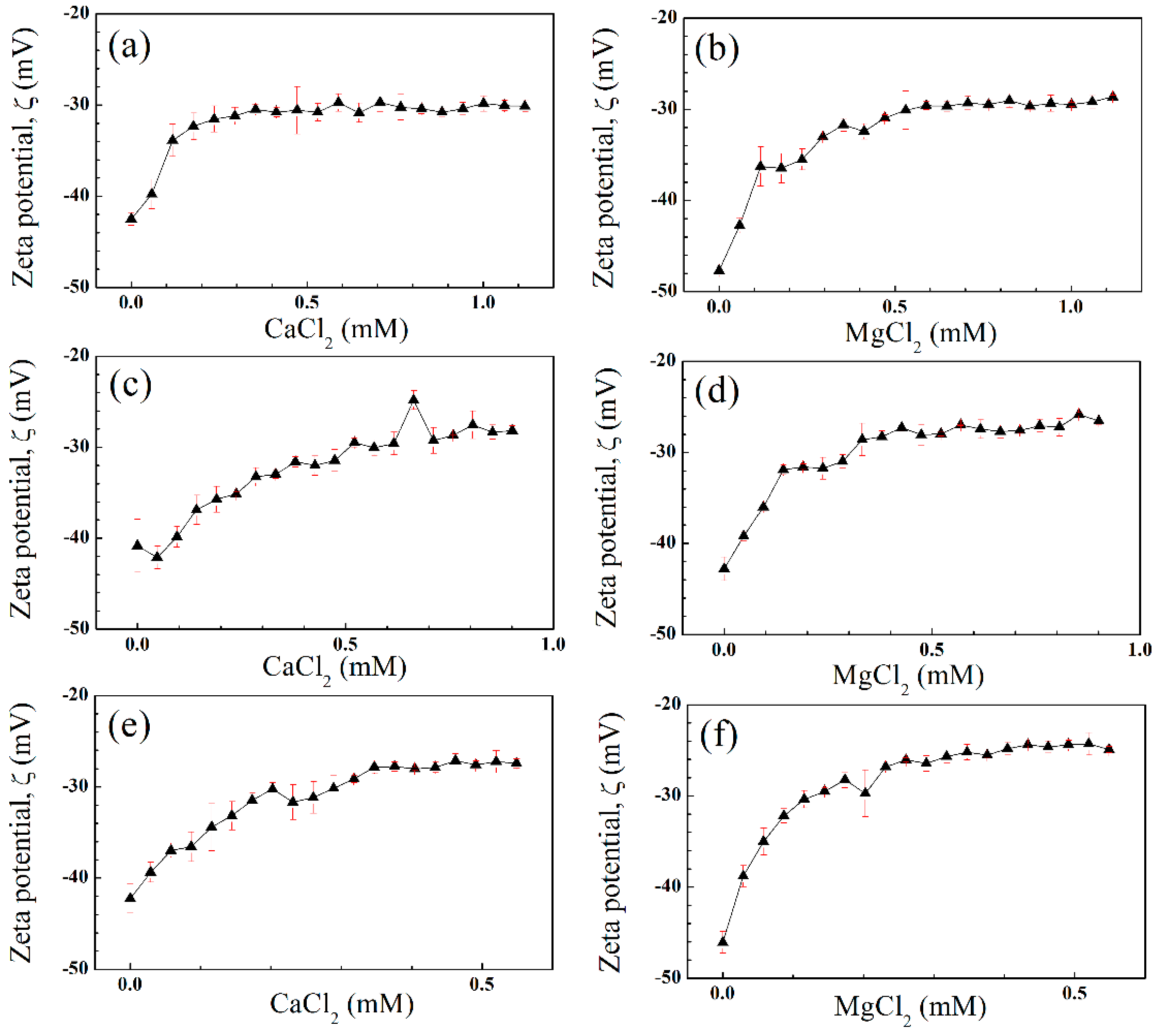

Figure S2. Zeta potential (ζ) of materials Poly(FA:CUR) for (a, b): 1 wt.%, (c, d): 3 wt.% and (e, f): 5 wt.% of chelating agents as function of concentration of calcium chloride ($CaCl_2$) and magnesium chloride ($MgCl_2$).

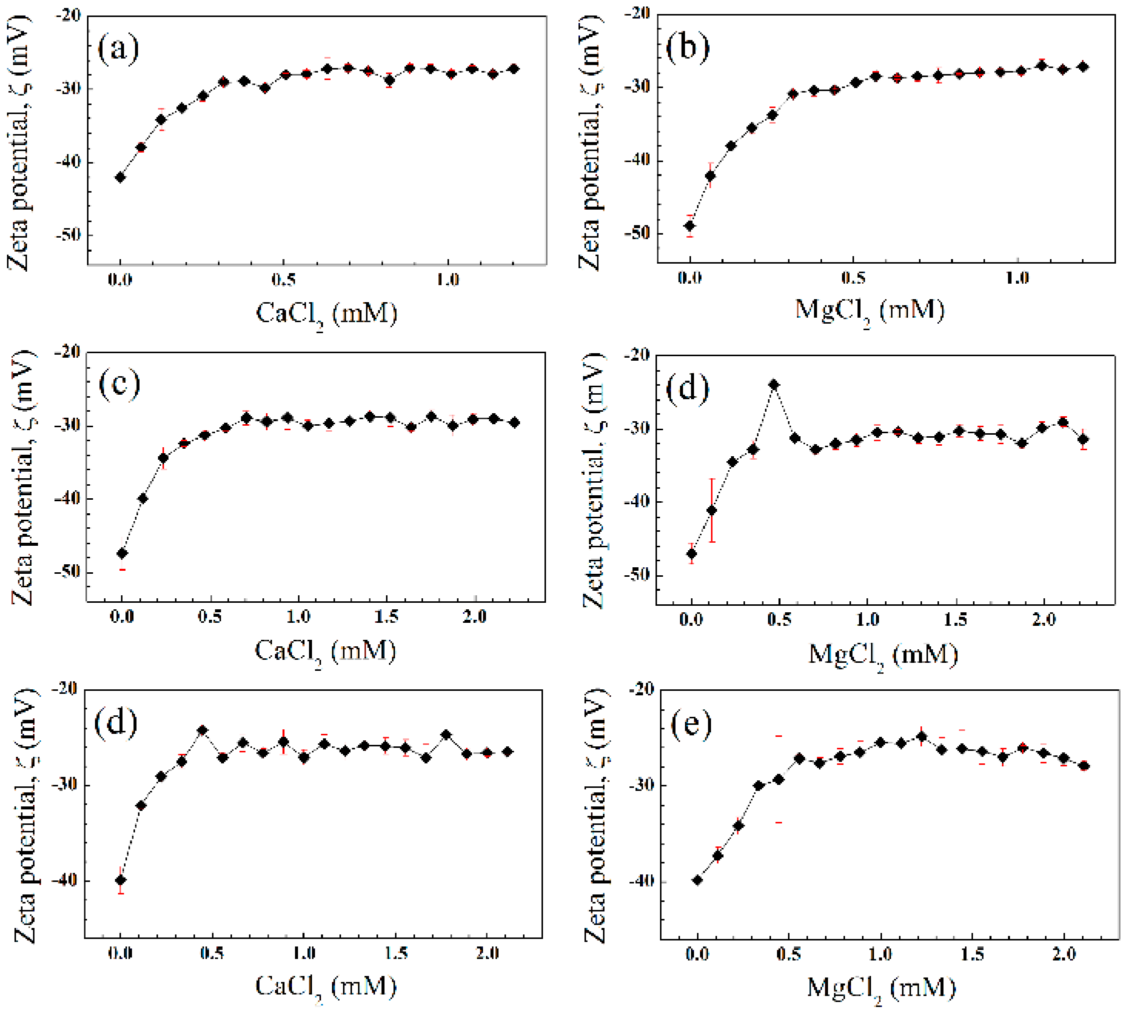

Figure S3. Zeta potential (ζ) of materials Poly(AA:FA:CUR) for (a, b): 1 wt.%, (c, d): 3 wt.% and (e, f): 5 wt.% of chelating agents as function of concentration of calcium chloride ($CaCl_2$) and magnesium chloride ($MgCl_2$).

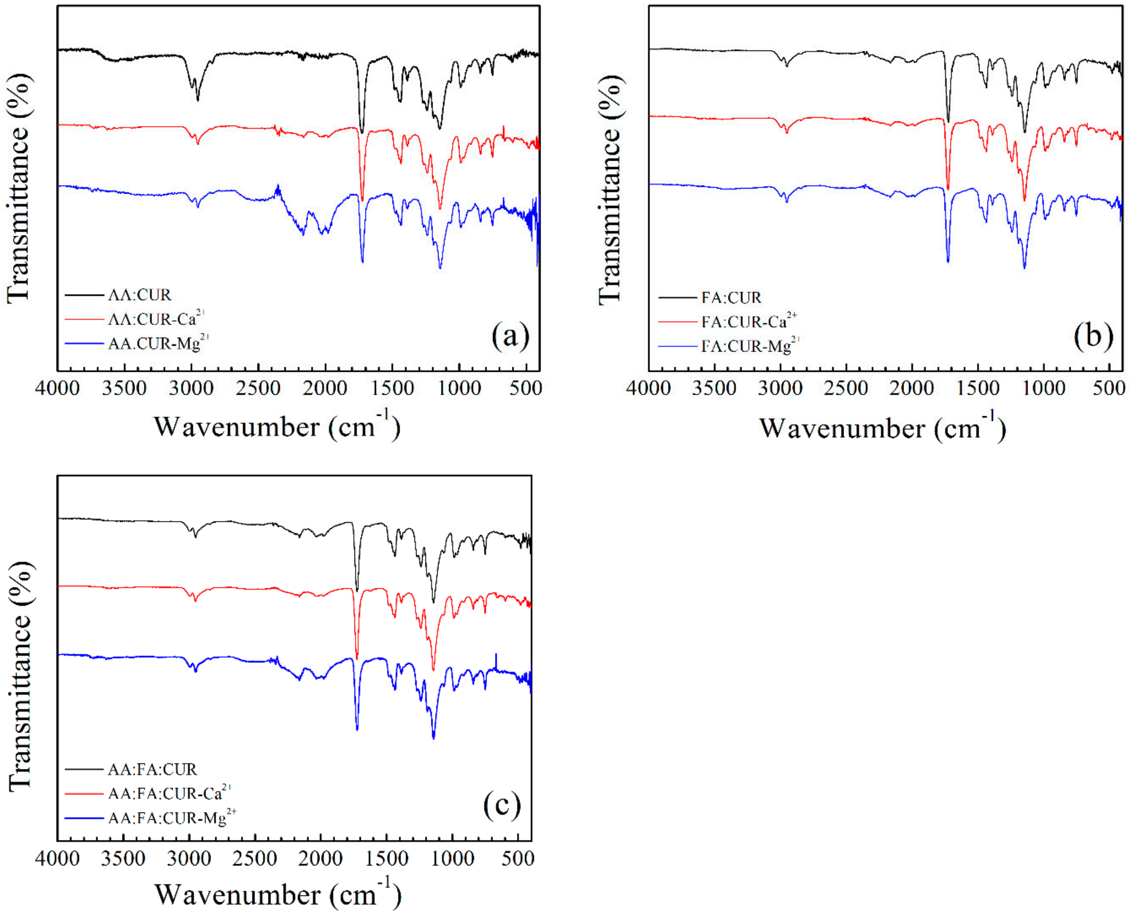

Figure S4. FT-IR Spectra of copolymers (a) Poly(AA:CUR)-Ca^{2+} and -Mg^{2+}, (b) Poly(FA:CUR)-Ca^{2+} and -Mg^{2+}, and (c) Poly(AA:FA:CUR)-Ca^{2+} and –Mg^{2+} systems.

Table S1. Average conductivity values of polymeric particles with 1 wt.%, 3 wt.% and 5 wt.% of chelating agents during the titration process with $CaCl_2$ by DLS technique, as example.

	**Conductivity (mS cm^{-1})**		
**Material**	**1 wt.%**	**3 wt.%**	**5 wt.%**
**Poly(AA:FA)**	0.282 ± 0.0514	0.630 ± 0.212	0.794 ± 0.177
**Poly(AA:CUR)**	0.2690 ± 0.0180	0.447 ± 0.125	0.480 ± 0.200
**Poly(FA:CUR)**	0.343 ± 0.0688	0.251 ± 0.0158	0.359 ± 0.137
**Poly(AA:FA:CUR)**	0.295 ± 0.0264	0.335 ± 0.0628	0.404 ± 0.0560
